# Complex Motor Learning and Police Training: Applied, Cognitive, and Clinical Perspectives

**DOI:** 10.3389/fpsyg.2019.01797

**Published:** 2019-08-07

**Authors:** Paula M. Di Nota, Juha-Matti Huhta

**Affiliations:** ^1^Department of Psychology, University of Toronto, Mississauga, ON, Canada; ^2^Office of Applied Research & Graduate Studies, Justice Institute of British Columbia, New Westminster, BC, Canada; ^3^Police University College, Tampere, Finland; ^4^Faculty of Education, University of Tampere, Tampere, Finland

**Keywords:** procedural learning, motor learning, plasticity, training, stress, physiology, occupational health, police

## Abstract

The practices surrounding police training of complex motor skills, including the use of force, varies greatly around the world, and even over the course of an officer’s career. As the nature of policing changes with society and the advancement of science and technology, so should the training practices that officers undertake at both central (i.e., police academy basic recruit training) and local (i.e., individual agency or precinct) levels. The following review is intended to bridge the gap between scientific knowledge and applied practice to inform best practices for training complex motor skills that are unique and critical to law enforcement, including the use of lethal force. We begin by providing a basic understanding of the fundamental cognitive processes underlying motor learning, from novel skill acquisition to complex behaviors including situational awareness, and decision-making that precede and inform action. Motor learning, memory, and perception are then discussed within the context of occupationally relevant stress, with a review of evidence-based training practices that promote officer performance and physiological responses to stress during high-stakes encounters. A lack of applied research identifying the neurophysiological mechanisms underlying motor learning in police is inferred from a review of evidence from various clinical populations suffering from disorders of cognitive and motor systems, including Alzheimer’s and Parkinson’s disease and stroke. We conclude this review by identifying practical, organizational, and systemic challenges to implementing evidence-based practices in policing and provide recommendations for best practices that will promote training effectiveness and occupational safety of end-users (i.e., police trainers and officers).

Law enforcement personnel including police officers rely on several types of information as they go about their duties and daily routines; external cues from the environment, internal physiology, declarative memory of laws and regulations, and implicitly learned tactical skills. Police are also entrusted to resolve potentially dangerous or violent encounters, in some cases necessitating the use of force. As a result, law enforcement personnel are exposed to high levels of occupational stress, which have been shown to pose risks to physical and mental health (Carleton et al., 2018, 2019; Planche et al., 2019). Policing skills, including physical capabilities and mental resiliency, are modifiable by training and experience and have an influence on police decision-making and performance in the field. To bridge the gap between empirical research and applied practice, we begin this review by describing initial learning processes (i.e., basic skill acquisition) before reviewing motor learning of specialized physical skills relevant to law enforcement. Specifically, we propose that situational awareness and decision-making are essential motor skills for policing that integrate sensory, motor, and cognitive functioning. The neurophysiological processes underlying procedural motor learning will be integrated throughout these discussions. Then, we show how occupationally relevant stress influences police performance, and has been adaptively integrated into state-of-the-art training to promote motor learning outcomes. Next, evidence from various clinical populations will be reviewed to identify cognitive and neurophysiological mechanisms that are important for procedural motor learning among police. Finally, we conclude our review by identifying practical, organizational, and systemic challenges to implementing evidence-based police practices and put forth recommendations to overcoming these challenges that will improve training effectiveness and direct future work.

Before we begin, the authors would like to emphasize that this review is not intended to criticize or condemn any current practices. Rather, the following review is intended to provide an accessible summary of what happens in the brain during complex motor learning (i.e., police training), as well as during real-world police encounters that induce physiological stress responses that directly influence whether training is recalled during in-the-moment decision-making. Our hope is that police trainers and curriculum developers will use this information to inform, update, or improve understanding of current training practices to maximize learning outcomes. As society, technology, and scientific knowledge continue to advance, so should police training practices for the purpose of maintaining public and occupational safety.

## Applied Motor Learning in Law Enforcement

The manner by which police officers learn motor skills is no different from other humans simply because of their occupation–they have to progress from initial skill learning to high proficiency using the same neurophysiological processes as experts in other domains. To acquire this expertise, police officers must undergo rigorous training and acquire experience in the field. In addition to learning specific motor skills such as firearm handling and hands-on tactics, police officers must also train visuomotor networks involved in situational awareness. Together with past experiences (in training and on the job) and individual action competencies, an officer’s perceptual assessments implicitly inform complex decision-making for choosing the most appropriate motor command during dynamic and unpredictable encounters. Neural mechanisms underlying effective police training methods remain unknown and will be inferred from fundamental science and research on clinical populations that experience breakdowns in the cognitive and neurological mechanisms that facilitate motor learning and memory.

### Early Motor Learning: Basic Competency and Novel Skill Acquisition

**Figure 1:**
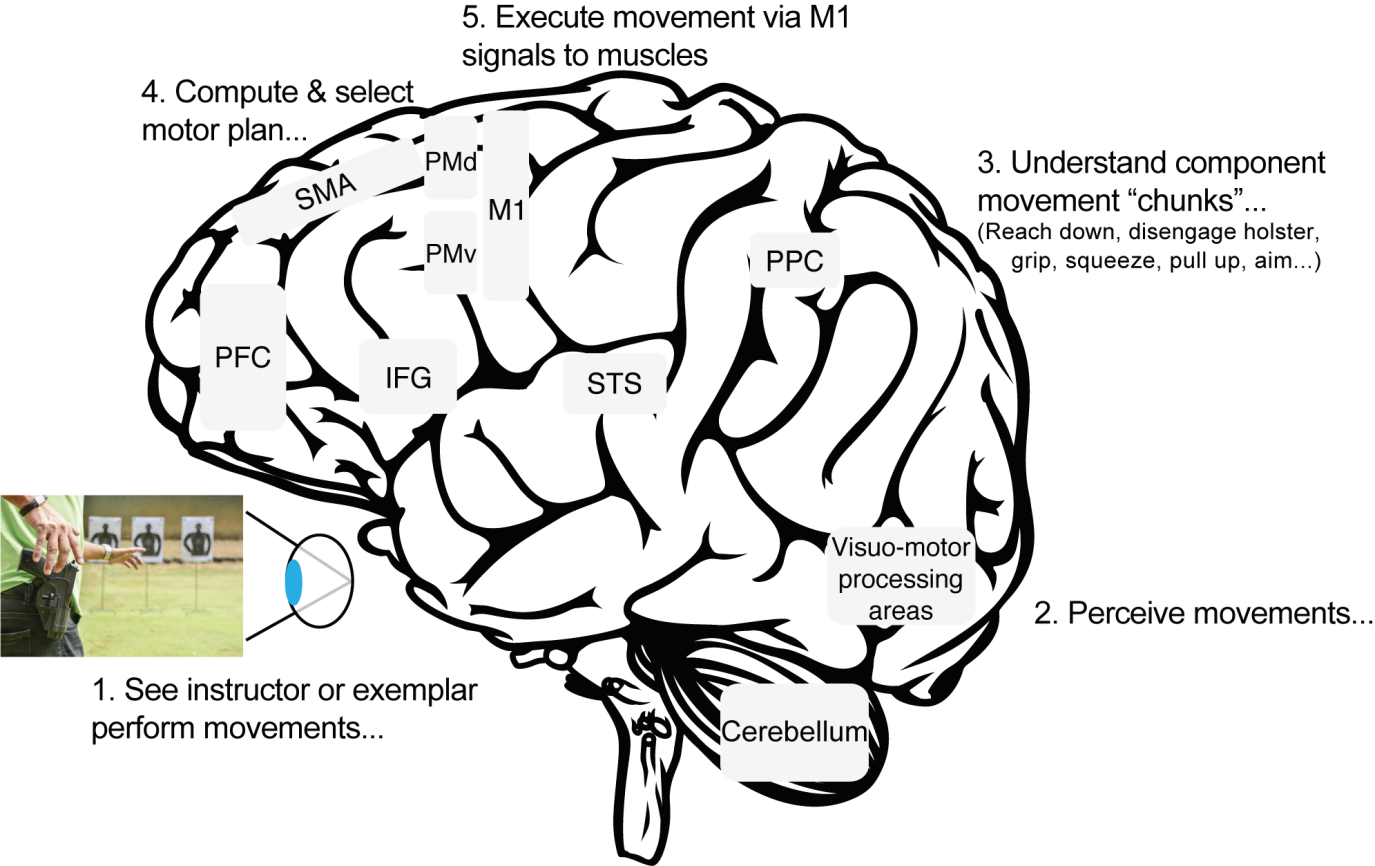
Transforming vision into action. Regions of the mirror neuron network (MNN) are independently responsible for a myriad of functions, including sensory perception, language processing, transforming external information onto internal body-centered reference frames, and planning and executing movements. Through repeated practice, the neuronal pattern of activation (or “motor representation”) underlying a novel movement is recalibrated and reinforced or consolidated into long-term memory. IFG, inferior frontal gyrus; M1, primary motor cortex; PFC, prefrontal cortex; PMd, dorsal premotor cortex; PMv, ventral premotor cortex; PPC, posterior parietal cortex; SMA, supplementary motor area; STS, superior temporal sulcus.

Skill acquisition and motor learning come with experience, which includes problem-solving through individual trial-and-error or during training with supervisors, teachers, or colleagues. Using firearm skills training as an example, new recruits (presumably without any prior experience with firearms) will carefully observe and model the behaviors of their instructor. A crucial step in the process of motor learning is the ability to define, understand, and remember the ordered sequence of observed movements (Figure 1). In order to do so, sensory-motor and memory regions of the brain are recruited to help break down continuous streams of motion (as well as music and language, see Zacks et al., 2009a; Francois and Schön, 2011; Lerner et al., 2011) into component “chunks” (Zacks and Sargent, 2010). Motor chunks begin and end with event borders that are typically marked by distinct kinematic movement parameters, including changes in position or location, speed, and direction of movement and also perceived changes in goals and intentions (Zacks et al., 2009b, 2010; Hemeren and Thill, 2011).

To continue with our previous example, drawing one’s firearm requires a set of discrete movements or chunks, including reaching down, releasing the gun from the holster, pulling the gun up from the holster to the chest and pushing it straight with the arms to the firing position, and aiming at a target. Segmenting motor sequences in this way facilitate early motor learning and gaining competence in smaller, more manageable units of information (Ericsson et al., 1980; Gobet and Simon, 1998; Bo and Seidler, 2009). With continued training, motor chunks can be grouped or “concatenated” into longer sequences (Sakai et al., 2003; Bläsing, 2015) that can be performed and recalled with less mental effort. Although it has not been directly investigated in police, one could hypothesize that experienced officers would identify fewer and larger chunks of component actions (e.g., a single motion for “pointing a firearm”) based on these previous findings. Future applied research on police segmentation behaviors would clarify the relationship between multisensory perception, motor learning, and memory. All of these cognitive processes are especially relevant for police, whose decision-making (i.e., motor selection) is guided by assessment of the environment, current physiological status (e.g., stressed, fatigued), and prior experience.

#### Reinforcing Motor Learning in the Brain

Our brains have evolved a highly sophisticated and complex network of brain areas that facilitate imitation-based learning. Giving credence to the old adage “monkey see, monkey do”, researchers unintentionally discovered the “mirror neuron network” (MNN, Figure 1) during neurophysiological investigations on reaching behaviors in monkeys (Gallese et al., 1996; Rizzolatti et al., 1996). Based on subsequent research in both animals and humans, the MNN has been shown to facilitate imitation-based learning by way of transforming observed or verbally instructed movements into a physically embodied action (for contemporary critical review, see Kilner and Lemon, 2013). Behavior is typically associated with the ability to execute a given movement but also involves observing and thinking about movement(s) through visualization or planning. That is, an officer’s MNN is activated while they are using their firearm, when they observe an instructor use their firearm, as well as when they visualize themselves using their firearm (Grèzes and Decety, 2001).

Based on common activation of the MNN during observation, visualization, and execution of movement, it begs the question: Can motor learning be achieved without physical practice? Several researchers have directly compared training gains across these paradigms for simple movement sequences (e.g., finger tapping). While similar performance gains (Hird et al., 1991), force gains, muscular motor-evoked potentials (Yue and Cole, 1992; Mattar and Gribble, 2005; Porro et al., 2007), and neural activation (Cisek and Kalaska, 2004) were found during observation- and visualization-based training paradigms, these measures and physical competency were less than movement-based training. Therefore, the neurophysiological connections enabling successful motor learning cannot be achieved to the same degree without physical practice^1^.

Brain regions comprising the MNN in humans include ventral and dorsal premotor cortices (Binkofski and Buccino, 2006), intraparietal sulcus, superior parietal lobe (Filimon et al., 2007), inferior parietal lobule, inferior frontal gyrus (Broca’s area), cingulate gyrus, cerebellum, superior temporal sulcus (Iacoboni et al., 2001), supplementary motor area (SMA), and primary motor cortex (M1). Individually, these nodes are functionally related to sensory, motor, language, attention, and memory processing (Figure 1). Together, the MNN transforms and maps externally perceived movement onto internal body-centered reference frames. Premotor cortices and SMA are primarily responsible for computing the desired motor plan, which is set into motion by triggering activation of M1 neurons that directly innervate corresponding muscle groups (for review, see Gallivan and Culham, 2015). There has been some debate regarding the activation of M1 and SMA during observation and visualization when motor output signals are inhibited and no overt movement occurs (Roth et al., 1996; Grèzes and Decety, 2001). Nonetheless, these nodes have shown reliable activation during all three types of movement processing to enable transformation of sensory and cognitive information into motor commands.

A given movement, such as drawing one’s firearm, is coded as a very specific pattern of neural activation in the MNN, referred to in the scientific literature as a “motor representation.” Once a given movement is performed (e.g., drawing, aiming, and firing a firearm), there is immediate visual feedback regarding whether the outcome was successful or not. These “incoming” visual signals, or *reafferents*, are compared to predictive “outgoing” *efference copy* signals that are generated by the brain during movement preparation (Blakemore et al., 1998; Rizzolatti et al., 1998; DeSouza et al., 2003). When predicted and actual movement is successful, and incoming feedback signals are congruent with the predictive outgoing signals, the motor representation is reinforced. Specifically, neural connections between MNN regions for the successful movement are strengthened, in turn facilitating future successful performance and engraining motor learning (Rizzolatti and Craighero, 2004; Oosterhof et al., 2013). When predicted and actual behavioral outcomes do not match, motor planning signals are recalibrated and updated with subsequent attempts, a process referred to as “motor adaptation” (Cressman and Henriques, 2009; Salomonczyk et al., 2011; Neva and Henriques, 2013).

In order to forge the functional connections between brain regions that code a novel motor representation, researchers have identified a competitive mechanism whereby stronger pre-synaptic inputs weaken the inputs from other neurons to the same post-synaptic cell, resulting in learning-dependent plasticity (Song et al., 2000). Made famous by Hebb (1949), neurons that “fire together, wire together”, known as spike-timing-dependent plasticity (STDP). In other words, the repeated and paired activation between neurons is reinforced with experience and training, forging stronger, and more reliable connections.

Just as training is intended to encode correct behaviors, it provides an opportunity to work through errors constructively (section “The Gold Standard for Complex Motor Learning for Police: Scenario-Based Training”). Our brains are equipped with specialized functions to ensure that those errors are not encoded over correct patterns of behavior. The precise timing of coordinated and long-range neural activation among regions of the MNN can induce states of anti-STDP, potentially blocking the encoding of new information that needs to be erased (Koch et al., 2013). For instance, anti-STDP processes could prevent the encoding of an officer’s incorrect drawing of their firearm or movement pattern through a training scenario that resulted in them being shot by an armed suspect. Break-downs in learning-dependent STDP mechanisms are observed in clinical populations, including individuals with Alzheimer’s disease (AD) and are discussed further in section “Seeing and Hearing is Believing: Superadditive Mechanisms of Multisensory Inputs”.

### Defining Expertise in Policing

#### Performance Enhancement

Initial motor learning is characterized by effortful practice and mastery of component actions or “chunks” (e.g., drawing, aiming, and firing) of larger action sequences (e.g., quickly reaching for one’s firearm). With continued training comes a reduction and eventual plateau in performance errors, reaction times, and the effort needed to execute now-automatized behaviors. Such performance measures have often been used to define expertise in empirical research studies of various problem-solving tasks. Expert knowledge is organized in large scale, multilevel, and interconnected data structures that integrate sensory, motor, and linguistic functions of component “chunks” of information (Di Nota, 2017). Increasing the number of chunks in novice thinking does not make him an expert but requires a structured organization of knowledge (Rauste-von Wright and Wright, 1994). As a result, experts are characterized to have an excellent ability to perceive the overall picture in different situations, with an unconscious understanding of how to meet the needs of novel situations (Ropo, 1991).

Defining expertise as a progressive linear process that encompasses a finite set of physical skills has been met with criticism. Several researchers argue that the extent or duration of training time is less important to defining expertise than an individual’s competence, with less experienced individuals outperforming experts in several domains (Doane et al., 1990; Vicente and Wang, 1998; Ericsson, 2004). One of the most prominent researchers in expertise is Ericsson, who proposed that the duration of training is positively correlated to improvements in performance that are tailored to typical situational demands. Once automaticity of behavior is achieved, additional experience will not significantly improve performance further or refine mediating neurological mechanisms, leading to arrested development (Ericsson et al., 1993; Ericsson, 1998). An appropriate example includes tying one’s shoelaces; once this skill has been mastered, additional experience will not be related to higher levels of performance.

To develop high-level skills, including those relevant to policing, Ericsson defines expertise as an ability to apply one’s skills adaptively to perform faster, more accurately, and with less effort under a wide variety of situational constraints and demands. Experts break through the ceiling of arrested development with deliberate practice, which involves effortful cognitive engagement in challenging tasks that may not commonly be encountered (Ericsson and Lehmann, 1996). According to this definition, experts attain higher levels of performance by challenging themselves to meet increasingly difficult demands, in turn developing a repertoire of increasingly complex motor representations. Ericsson’s theoretical framework is especially relevant for police who train for highly dynamic, uncertain, and potentially dangerous encounters. Section “Bridging the Gap Between Science and Practice: Evidence-Based Police Training” will review the current state of the art for police training paradigms that consider the principles of deliberate practice, as well as the influence of physiological responses to occupationally induced stress, to promote motor learning and effective recall during critical incidents.

Through the overt (sensory reafferents) and covert (efference copy) feedback processes described above, expert sensorimotor networks facilitate decision-making, performance, and novel motor learning that is faster and more accurate than among novices. The refinement of complex networks that encompass sensory, motor, language, and cognitive (i.e., memory, decision-making) brain regions suggest a high potential for skill transfer across domains and bear important implications for the therapeutic application of motor learning and training for a variety of disorders (see section “Therapeutic Benefits of Complex Motor Learning”).

#### Situational Awareness

By their very nature, high-stakes police encounters are highly complex and always changing. Among police instructors, it is understood that motor skill learning in and of itself is not sufficient to cope with the complex reality of police encounters. An example would be a situation in which the police have the conditions and necessity to use a firearm toward a target person, but there are many bystanders in the vicinity. In this case, using a firearm could be a serious threat to public safety. In addition to the basic motor competency and handling of the weapon, the officer must also be able to assess and change their positioning effectively so that discharging the firearm can minimize collateral damage and effectively resolve the situation. If an officer lacks knowledge (and training) in situational awareness and decision-making, the outcome of highly unpredictable, time-pressured, and stress-inducing encounters like the one described here is likely unfavorable. Therefore, situational awareness and subsequent selection of the best course of action are fundamental procedural skills for police that inform behavioral outcomes just as much as basic motor learning.

Although the conditions and circumstances to every situation are unique, police instructors and practitioners generally agree that situations can be understood as a whole, within which there are fundamental elements that can be separately understood and trained. An officer’s perception and evaluation of a situation directly informs what motor skills they will employ. This online assessment of the environment is known as situational awareness. Several definitions of situational awareness exist for different fields but has been defined by Endsley (1995) as possessing three components: perception, comprehension, and projection. In other words, sensory perceptions signify elements of the environment, whose meanings must be understood in order to anticipate their future status in relation to the objectives of the action. Before we are able to understand our perceptions, it is important that we learn to make proper perceptions. Selective attention is an important function of the sensory system because all of the information received by the senses cannot be consciously perceived at the same time (Tiippana, 2006). Therefore, what part of the external environment is the subject of conscious awareness at any given time is controlled by attention, which is highly influenced by stress (see section “Stress-Induced Memory Deficits, Perceptual Distortions, and Performance Errors in Police”). According to this view, selective attention divides the external totality of a situation into meaningful and non-meaningful elements, the latter of which is ignored and the remaining essentials are attended (Varila and Rekola, 2003).

In the case of training aimed at developing situational awareness, it would be advisable to develop methods of visual exploration and subsequent processing of critical information (Salas et al., 1995). Once essential features of the environment have been identified, complicated situations can be broken down into smaller elements or “chunks.” Just as with fundamental motor learning described above, situational awareness training can afford novices the opportunity to recognize chunk patterns in different contexts and combinations, and link them to appropriate motor strategies (Varila and Rekola, 2003). As shown by previous research (Bläsing, 2015), police experts may sum up several observations into larger entities that include both situational awareness and tactical elements. Indeed, an examination of police shooting strategies found significant overlap in stepping and shooting behaviors (Nieuwenhuys et al., 2017), reflecting concatenation of component motor and perceptual chunks during a high-threat shooting exercise. Without investigation, standardization, and validation of situational awareness training strategies, police officers may be learning wrong patterns and encoding stimulus-response tendencies instead of effective critical thinking skills.

#### Fast, Flexible, and Accurate Decision-Making

In both policing and basic science, actions are typically evaluated by the final outcome. In reality, human behavior is far more complex than a hierarchical, step-wise process that begins with a goal, is followed by a conscious motor plan, and concludes with an appropriate movement. Researchers in the field of computational neuroscience have provided an alternative school of thought that suggests multiple behavioral outcomes, or “affordances,” unconsciously competes for final selection (Cisek, 2006, 2007). Based on current perceptions of the environment, the brain considers multiple potential motor affordances to achieve a desired outcome. For instance, a suspicious individual in a dark alley may elicit multiple behaviors from an officer, including verbal commands, change in positioning, and accessing one of multiple force options [e.g., baton, oleoresin capsicum (OC) spray, conducted electrical weapon, firearm]. As the situation unfolds over time, goals and available options for action selection are continuously updated by the prefrontal cortex (PFC) and basal ganglia, respectively.

In the current example, the suspect could charge toward the officer with a weapon necessitating a use of lethal force, or the suspect may comply with officer’s verbal commands and allow for safe approach. Cisek’s (2006, 2007) model suggests that updated sensory information biases competition among multiple motor affordances toward a single response that is released into execution. Further, there is evidence to suggest that high levels of threat narrow perceived and actual motor affordances for possible action (Pijpers et al., 2006). We propose that complex decision-making undertaken by police officers during high-stakes encounters involves several other factors, including stress-induced perceptual biases and prior experience acquired through training or in the field. These considerations and their unconscious influence on police performance and motor selection will be discussed in detail in section “The Influence of Stress on Police Performance.”

Once motor learning is engrained, and officers are adequately trained in situational awareness, how is this knowledge functionally used “in the moment”? Based on acquired knowledge from training and work experiences, officers make well-informed decisions very quickly under conditions of extreme time pressure, high stakes, and shifting conditions (Klein, 2017). However, they may not be able to describe how or why they chose to act (Ropo, 1991). Decades of investigations with experts in several fields, including emergency first responders and military personnel, support two prominent theories that characterize “intuitive” decision-making. In contrast to deliberate, slow, and controlled reasoning, Kahneman and Tversky’s Two-System Model (Stanovich and West, 2000; Kahneman, 2003) stipulates that intuitive decision-making is automatic, effortless, and not available to introspection. Often emotionally charged, intuitive decision-making elicits habitual responses that are difficult to control or modify, highlighting the importance of cementing correct (or optimal) intuitions with police training.

Klein’s Recognition-Primed Decision Model (RPDM) (Klein, 1989, 1993) characterizes proficient decision-making as a fusion of two mental processes – situational awareness and mental simulation (or visualization). Experienced officers recognize familiar cues and patterns of information in the environment and quickly identify what goals and actions are feasible or not. In contrast to competitive affordance models (Cisek, 2006, 2007), the RPDM stipulates that there is no concurrent deliberation of alternate options. Rather, a single action plan that is most likely to meet a sufficient outcome is mentally simulated. If any pitfalls are expected, the action plan is adjusted until a satisfactory outcome is realized and executed. Because there is no deliberation, decision makers often cannot explain their rationale.

The difficulty of articulating implicit decision-making also poses a problem for training and evaluation of police motor learning, which is largely outcome based. Steps should be taken to ensure that the physical tactics and cognitive thought processes leading up to (and including) the decision to act are adequately addressed in police training. Experts are also shown to make “rookie mistakes” when ignoring relevant cues for the sake of fast decision-making (Kahneman, 2003). Through more introspective pedagogical approaches, police trainers can use mistakes in both novice and expert officers’ performance to recalibrate and reinforce correct intuitive cognitive and motor strategies.

#### Confidence and Action Competency

Finally, we suggest that the practical application of procedural motor skills, situational awareness, and expert decision-making might also be linked to the officer’s individual perception of their own skills and abilities that precedes action. As reflected in military pedagogy, the concept of action competence refers to one’s self-perceived ability to act, which includes physical (i.e., operational) and mental capability, competence, knowledge, and skills that are essential to an individual’s survival in demanding situations (Toiskallio and Mäkinen, 2009). Action competence can also be defined with respect to social and ethical considerations, including ownership and justification of one’s actions that undoubtedly influence police and law enforcement performance. Action competence is another aspect of complex motor learning that trainers and curriculum developers should be aware of when reimagining police training methods and approaches.

#### Changes to Brain Structure and Function With Long-Term Training and Expertise

Investigations of motor learning are typically examined in highly controlled experimental settings using simple tasks, including arm reaching, finger tapping, and eye movements. However, the physical skills of police and other law enforcement personnel require complex, whole-body movements that are highly dynamic and dependent on the unique situation at hand. To investigate learning-induced neural plasticity that is more applicable to real-world experiences, we look to other areas of research including sport psychology of athletics, dance, and music. These domains have served as ideal models for measuring ecologically valid, reproducible, sequential movements that have established standards for correct performance. Neurological evidence for motor learning among police is a largely unexplored area of study (see section “Live Versus Virtual Scenario-Based Training”), and the few empirical research studies investigating training-induced changes to police physiology (i.e., cardiovascular) and performance will be reviewed in section “The Gold Standard for Complex Motor Learning for Police: Scenario-Based Training.”

To facilitate dynamic expert performance, neuroimaging findings of long-term training have shown greater structural organization and neural efficiency among brain regions involved in motor planning among experts relative to novices. Specifically, expertise has been linked to reduced gray matter volume in superior frontal gyrus, left PMC, SMA, and putamen relative to non-experts, and lower white matter volumes in bilateral corticospinal tracts and corpus callosum (Hänggi et al., 2010). Fractional anisotropy, which measures the extent of fiber integrity (Assaf and Pasternak, 2007), is also lower in white matter tracts underlying PMC among experts, reflecting less diffusion across white matter tracts (Hänggi et al., 2010).

Reorganization of expert brain networks also facilitate faster and less effortful learning of new information related to one’s area of expertise (Shmuelof et al., 2012), lending empirical evidence to the old adage that “you can teach old dogs new tricks.” Brain imaging studies of novel sequence learning among longstanding experts show initial increases in neocortical activation (SMA), reflecting effortful cognitive motor planning. Once the motor sequence has been automated and over-learned (i.e., practiced daily for several weeks with a high degree of accuracy), there is a dramatic decrease in neocortical activation and greater activation in subcortical regions including the striatum (Bar and DeSouza, 2016). The striatum is a critical part of the brain’s motor and reward systems, is reciprocally connected to the PFC and thalamus, and coordinates numerous cognitive functions including action planning, decision-making, motivation, and goal/reward processing (Yager et al., 2015). These connections enable optimal expert performance and involve processes that directly inform police decision-making as discussed above. Despite the body of neuroimaging evidence reviewed here, there is a dearth of investigations examining training-induced changes to brain structure and function among police (see section “Future Directions for Evidence-Based Police Training: Neurophysiological Mechanisms” for current evidence and future directions).

## The Influence of Stress on Police Performance

Despite the comprehensive overview of occupationally relevant motor learning presented above, an important problem for police training remains: how can we promote recall of training during high-stress, time-limited encounters in the real world? By examining how physiological responses to stress influence police performance, perception, learning, and memory, we provide a framework for understanding effective training programs that use evidence-based principles to prepare police officers for the realities of the frontline.

### Physiological Responses to Stress

The most scrutinized decisions made by police officers usually occur under highly stressful conditions resulting in a use of force and particularly lethal force. Despite expectations to perform in accordance with the law and their training, law enforcement personnel and other first responders are not immune to the body’s automatic physiological responses to threat and stress (for detailed description, see Schwabe and Wolf, 2013; Ness and Calabrese, 2016). By initiating the “fight or flight” response, stress adaptively promotes survival by mobilizing individuals to escape threat (Lovallo, 2016). Stress can be measured by various objective physiological markers. Cardiovascular indexes of stress include heart rate (HR), heart rate variability (HRV), blood pressure, and galvanic skin response. Cortisol obtained through saliva or blood samples provide a neurochemical measure of stress. Very little research has been conducted on police using non-invasive brain imaging techniques, including magnetic resonance imaging (MRI) or electroencephalography (EEG) (see section “Future Directions for Evidence-Based Police Training: Neurophysiological Mechanisms”). Both neuroimaging techniques stand to illuminate structural and functional changes to the brain following acute and long-term exposure to stress, as well as learning-induced plasticity.

**Figure 2:**
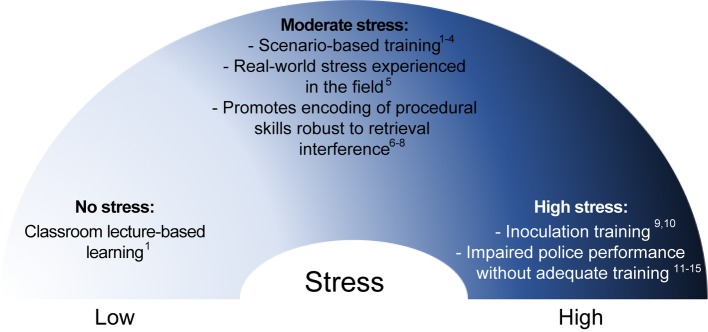
The stress-memory continuum. As established by Yerkes and Dodson’s (1908) seminal work, stress influences learning and memory processes on an inverted U-shaped continuum. At moderate levels, stress promotes attentional arousal and encoding (i.e., learning) of novel information. At extremely high levels, stress interferes with both encoding and retrieval processes and is maladaptive in training contexts. Based on empirical physiological evidence from police officers, scenario-based training lies at an optimal position of the continuum whereby ecologically-valid levels of stress are induced and result in measurable improvements in performance, including use of force decision-making. ^1^Andersen et al., 2016a; ^2^Andersen and Gustafsberg, 2016b; ^3^Andersen et al., 2018; ^4^Armstrong et al., 2014; ^5^Anderson et al., 2002; ^6^Jameson et al., 2010; ^7^Cahill and Alkire, 2003; ^8^Cahill and McGaugh, 1998; ^9^Morgan III et al., 2004; ^10^Taverniers et al., 2013; ^11^Oudejans, 2008; ^12^Nieuwenhuys and Oudejans, 2010; ^13^Nieuwenhuys et al., 2012a; ^14^Nieuwenhuys et al., 2009; ^15^Nieuwenhuys et al., 2012b.

Seminal work by Yerkes and Dodson (1908) has been supported by basic and applied research in several fields to establish the stress-memory continuum (Figure 2), which demonstrates that low to moderate levels of stress “arousal” adaptively facilitate learning, memory, and cognitive performance (Jameson et al., 2010). The strength of a memory is proportional to the level of arousal it elicits (Thayer and Sternberg, 2006), with stronger encoding of new information with more robust stress responses. However, at extreme levels, stress is maladaptive for learning by blocking both encoding of novel information and memory retrieval.

Basic science and animal research shows that the precise timing of stress-induced release of neurochemicals is key to successful encoding of novel information. Improved learning outcomes are observed when epinephrine is administered immediately prior to or during learning (Cahill and McGaugh, 1998; Cahill and Alkire, 2003), and severe impairment in memory recall is observed when glucocorticoids are administered before retention testing (De Quervain et al., 1998, 2000; Diamond et al., 2006). These findings reveal unique influences of the various neurochemicals released during human stress responses on learning (i.e., memory formation). An important consideration for developers of police training programs is to identify an optimal level of stress that adaptively promotes learning without crossing the threshold for maladaptive stress that interferes with encoding and retrieval processes.

### Stress-Induced Memory Deficits, Perceptual Distortions, and Performance Errors in Police

There is a limited yet growing body of research investigating the effects of stress on police performance, learning (see section “Bridging the Gap Between Science and Practice: Evidence-Based Police Training”), and memory (e.g., Yuille et al., 1994; Stanny and Johnson, 2000; Hope et al., 2016; Lewinski et al., 2016), with several insights offered from studies on military personnel (Morgan et al., 2004, 2006; Taverniers et al., 2013). These studies tend to focus on declarative memories of extremely stressful training and work situations, including prisoner of war exercises and police-involved shootings. Results consistently show stress-induced impairments to both immediate and delayed memory. For instance, officer recall of their path of travel during a simulated high-stress traffic stop significantly deviates from their actual path of travel, highlighting the influence of stress on officers’ spatial memory (Lewinski et al., 2016). These outcomes bear greatly on the accuracy of police or eyewitness memory of traumatic encounters, as well as recall accuracy during stressful questioning procedures like evaluations, inquests, and trial proceedings. Considerations of stress on police memory lie beyond the scope of the present review (see Hope, 2016), which aims to clarify the influence of stress on police motor learning and performance.

Qualitative evaluation of police officers’ accounts of encounters where they shot citizens reveal several consistent perceptual distortions, including diminished sound, slowed time, tunnel vision (i.e., narrowed attention), and heightened sense of visual detail (Klinger and Brunson, 2009). Even though officers are inherently aware of the temporal dynamics of action-reaction, they have been shown to systematically underestimate the distance between themselves and suspects in both low- and high-threat conditions (Nieuwenhuys et al., 2012a). In fact, research showing that an officer already pointing their gun at an armed suspect is unable to fire before the suspect does (Blair et al., 2011). These natural, untrained tendencies can lead to devastating outcomes unless stress-induced perceptual distortions are also considered and integrated into training procedural motor skills relevant to police (see section “Bridging the Gap Between Science and Practice: Evidence-Based Police Training”).

Based on the significant overlap of brain networks involved in movement, learning, attention, and physiological stress responses, several psychological theories offer possible mechanisms for stress-induced impairments in police performance. Where an officer directs their attention informs their perception, evaluation, and available behavioral options for motor selection. To enable fast decision-making, the brain predictively “sees” before conscious perception (Barrett and Bar, 2009; Barrett, 2012). This phenomenon is known as affective realism, whereby external stimuli are assigned an emotional or affective “value” (i.e., gun = bad) that informs downstream physiological processes to approach or avoid the stimulus. As such, reporting negative emotions can increase the likelihood that a benign object like a wallet or cellphone is visually perceived to be a gun (Baumann and DeSteno, 2010). While this type of perceptual distortion rarely contributes to misinterpretation in violent police encounters, it has been reported in the past (e.g., 1999 police shooting of Amadou Diallo in New York City). An empirical research study on military cadets found faster and more accurate identification of a weapon versus a tool when visually primed with a threatening image but also an increase in “false positives” for weapons when presented with a tool under high anxiety conditions (Fleming et al., 2010). Training that integrates realistic levels of stress can help promote accurate perceptions over false positives that bear significant implications for occupational safety and security.

Applied research on police shooting performance has shown greater stress (self-report and HR), faster reaction times, and decreased shooting accuracy during high-threat conditions, where a live actor or canon shoots back at officers with simulated ammunition, versus low-threat conditions where officers shoot a static target (Oudejans, 2008; Nieuwenhuys and Oudejans, 2010; Nieuwenhuys et al., 2012b). Stress-induced decrements in police performance have also been found for other complex procedural skills, including arrest and self-defense behaviors typically used in the field (Nieuwenhuys et al., 2009). According to attentional control theory (ACT), a stressful or threatening stimulus exerts both negative and positive influences on attention, respectively, by: (1) drawing attention away from task-relevant information toward distracting threat-relevant information and internal worries, leaving fewer attentional resources to effectively perform the task at hand and (2) increasing motivational cognitive or mental effort on task performance to counteract negative attentional effects (Eysenck et al., 2007).

Consistent with ACT principles, head and eye tracking reveal increased attention toward the suspect (i.e., threat) and away from task-relevant targets. Increased motivation is supported by higher reported mental effort during high-threat conditions, as well as faster reaction times to eliminate the threat but at the cost of shooting accuracy (Oudejans, 2008; Nieuwenhuys and Oudejans, 2010; Nieuwenhuys et al., 2012a; for discussion on conflicting attentional mechanisms, see Hope et al., 2016). Similar to Fleming et al. (2010), Nieuwenhuys et al. (2012b) found a greater bias toward shooting unarmed targets in high-threat conditions, in addition to faster reaction times and decreased shooting accuracy to armed targets. Despite sampling highly trained police officers, the high-threat condition in these investigations influenced subconscious attentional and motivational processes that superseded officers’ training to respond to a threatening situation, impairing task-related shooting performance. These findings highlight the urgent need to address occupationally relevant stress during police training to mitigate impairments in perception and subsequent decision-making and performance.

### “I Don’t Feel Stressed”: Subjective Versus Objective Measures of Stress

Further contributing to the nuanced and highly complex relationship between stress, learning, and memory is the fact that stress is a highly individual experience. What may be perceived as extremely stressful for one individual may not be stressful at all to another. One’s perceived level of stress may also differ from objective physiological measures, especially among police and law enforcement personnel who may be hesitant to admit “feeling stressed.” Physiological stress responses in police have been shown to manifest in very similar ways during an encounter (e.g., physical use of force interactions) or in anticipation of an encounter (e.g., driving to event with lights and sirens; hand on gun) (Anderson et al., 2002). Stress can also be triggered by “real” external cues in the environment (e.g., presence of a lethal weapon) or by internal psychological states (e.g., fear or anticipation of observing a lethal weapon), further complicating the investigation of how stress impacts police performance. Recent evidence shows that law enforcement personnel have significantly higher baseline levels of cortisol relative to the general population, and that tactical officers exposed to greater occupational threat have even higher levels of cortisol than frontline police officers (Planche et al., 2019), bearing greatly on the long-term health trajectories for individuals in high-risk occupations. Thus, individual and occupationally mediated differences in stress responses confound the determination of where any single police officer (or individual) lies on the stress-memory continuum (Figure 2), and what level of stress meets the threshold for maladaptive arousal. Objective measures of behavior and physiological stress such as HR, HRV, and salivary cortisol are crucial in evaluating the true influence of stress on individual performance.

## Bridging the Gap Between Science and Practice: Evidence-Based Police Training

In recent years, there has been significant development and progress in the field of evidence-based policing, which uses empirical research to validate the effectiveness of various training approaches. The most studied training, and relevant for our discussion on complex motor learning, is use of force training. Most investigations of police behavior examine firearm use (i.e., shoot/no-shoot decisions), accuracy, and timing, but a use of force can range from physical (i.e., hands-on) tactics to any tools available to police officers including baton, OC spray, conducted electrical weapon, or firearm. In addition, we have proposed that situational awareness and decision-making are essential procedural motor skills for effective policing that are also influenced by occupationally relevant stress. The following section will review the current state of the art in evidence-based training that attempts to find a balance in the stress-memory continuum (Figure 2) and promote effective motor learning for police.

### The Adaptive Role of Real-World Stress in Police Training

Through occupational experience, police officers can learn to adapt and overcome the negative influences of stress on perception and performance described above. A study comparing novice and expert police officers found improved shooting behavior and gaze control in the expert group under high-threat conditions (Vickers and Lewinski, 2012). Police officers who have better regulation of their stress responses have been shown to use the associated physiological cues in an adaptive way to promote performance (i.e., fewer shooting errors, de-escalating potentially violent encounters) (Akinola and Mendes, 2012; Haller et al., 2014). These findings suggest that increased exposure to, and familiarity with, occupationally relevant stress can offset its interfering effect on performance.

In a series of investigations on athletes and police officers, Oudejans and colleagues have established efficacy for performance training that integrates occupationally relevant stress. Beginning with basketball and darts players, Oudejans and Pijpers (2009, 2010) found that training with mild levels of anxiety improved post-training performance under stressful conditions compared to control groups that did not train with stress and who showed stress-induced deterioration of performance. Expanding on the police performance studies mentioned previously (Oudejans, 2008; Nieuwenhuys and Oudejans, 2010; Nieuwenhuys et al., 2012a,b), Oudejans and colleagues examined the efficacy of training police officers in high-stress (with a live actor or canon shooting back simulated ammunition) and low-stress (officer shoots at static target or mannequin) conditions. Officers completed pre- and post-training tests on firearms use of force performance under high- and low-stress conditions. All officers showed increased HR, poorer shooting accuracy, greater mental effort, and greater attentional fixation on threat- (versus task-) relevant stimuli during high-stress pre-training evaluations. Officers trained under high-stress conditions no longer showed impaired shooting performance during stressful post-training evaluations (Oudejans, 2008) or at 4-month follow-up evaluations (Nieuwenhuys and Oudejans, 2011).

Similar to the principles of motor adaptation described earlier in the section on “Applied Motor Learning in Law Enforcement”, the researchers suggest that training-induced performance gains are facilitated by recalibration of officers’ selected motor plans (Nieuwenhuys and Oudejans, 2011). Even though officers still exhibited physiological stress responses post-training, their performance improved as a result of training under the same high-stress conditions in which they were expected to perform. Further, there is a relationship between stress and an officers’ motor selection strategy, such that inhibiting a preferred motor plan (e.g., shooting with a grounded stance) due to situational constraints results in greater reported anxiety and reduced shooting accuracy, even if the preferred motor plan is slower and puts the officer at greater risk (Nieuwenhuys et al., 2017). Therefore, integrating stress into a repetitive training paradigm not only promotes police performance during subsequent high-stress conditions but also facilitates motor learning that can override preferred movement strategies that would put the officer at risk.

### The Gold Standard for Complex Motor Learning for Police: Scenario-Based Training

Consistent with scientific principles of motor learning (section “Applied Motor Learning in Law Enforcement”), police trainers generally agree that basic skills training should begin with learning the fundamentals, or component “chunks”, in order to develop proficiency, comfort, and safety with a given technique (Pryor, 1999). Once motor skills are overlearned and deeply encoded in long-term “muscle memory,” training approaches must evolve in order to ensure the motor skill can be flexibly applied to a variety of stressful circumstances that necessitate a use of force. Surveys from experienced police trainers (Aldred, 2017) and a growing body of empirical research support the efficacy of scenario-based training, which simulates the stress and complexity of real-world situations to a greater extent than classroom lecture-based learning or static drills (Armstrong et al., 2014; Andersen et al., 2016a).

Scenario-based training is fully immersive, utilizing real and artificially constructed environments (e.g., schools, communities, and housing complexes), props, sounds, and lighting to create realistic environments that require various behavioral strategies. Professional actors or experienced police instructors are used to role-play various types of encounters ranging from violent offenders to domestic disputes and individuals in psychological crisis.

In accordance with Ericsson’s framework for deliberate practice (Ericsson and Lehmann, 1996; Ericsson, 1998, 2004), scenario-based training promotes expert motor learning among police by engaging the following principles:

1. Once police officers acquire proficiency through basic skill training, expertise is developed through exposure to increasingly complex and demanding situations.
2. By affording officers the opportunity to “feel” the physical sensations that accompany high-stress encounters, arousal-based mechanisms promote encoding of the learning experience and also allow officers to work through the stress response to achieve outcomes.
3. Trainees are afforded integrated practice of verbal, physical, and cognitive (i.e., decision-making, situational awareness) skills, building a repertoire of varied experience that increases the likelihood that skills will be generalized to other situations (Barney and Shea, 2007).
4. With constructive and immediate feedback from qualified instructors, multiple behavioral options can be explored for successfully resolving an encounter through discussion and mental simulation. This is especially beneficial to novices (i.e., new recruits) that can work through errors and dangerous encounters in a safe environment (see section “Reinforcing Motor Learning in the Brain” for anti-STDP mechanism for erasing unwanted information).
5. Deliberate practice through repeated attempts or trials reinforces the neural pathways of mental and physical skills (section “Reinforcing Motor Learning in the Brain”).
6. Training is administered at an appropriate level of difficulty to challenge the learner but not to ensure failure (i.e., no-win situations) or be too easy.

Very few studies have investigated the efficacy of scenario-based training on police performance or attitudes but show significant improvements after even a single day of training. Krameddine et al. (2013) administered a 1-day scenario-based mental health training program and found significant improvements in verbal de-escalation, communication, and empathy with the public up to 6 months post-training. With respect to procedural use of force training, Andersen and Gustafsberg (2016b) and Andersen et al. (2018) have shown immediate and long-term efficacy of a 4-day performance and resilience program (iPREP). Officers use real-time HRV biofeedback (HRV-BF) during immersive live-action scenarios to modulate their individual stress responses and promote recovery from threat by engaging adaptive peripheral nervous system dominance (Thayer and Sternberg, 2006; Lehrer and Gevirtz, 2014). Officers condition adaptive autonomic stress responses through repeated practice of various breathing techniques and mental simulation during various scenarios that increase in complexity and stress. Investigations of special forces and frontline officers have shown significant reductions in use of force shooting errors, improved situational awareness (Andersen and Gustafsberg, 2016b), and faster autonomic recovery to baseline following stressful training scenarios up to 18-month post-training (Andersen et al., 2018).

By integrating pure motor skill practice with realistic environments, stress, and various decision-making options and outcomes, scenario-based training ensures cognitive motor skills (i.e., situational awareness, decision-making, visualization, breathing techniques) are adequately encoded and reinforced in learning and memory systems.

#### Mental Skills Training

The “mental skills” engaged during iPREP, including visualization and stress-reducing breathing techniques, are physically conditioned (i.e., become implicit and are performed without conscious effort) using HRV-BF. Other police training interventions that incorporate mental skills have shown efficacy in improving use of force performance, reported and objective (HR) measures of stress, and negative mood during high-stakes scenarios (Arnetz et al., 2009). However, this training paradigm spanned 10 weeks, and post-training evaluations were conducted 12 months later. Therefore, it is unclear whether significant findings are due to the specific training intervention or 1 year of training and field experience.

Another investigation on the efficacy of two 75-min breathing, imagery, and attentional control training sessions found improved memory for details during a stressful OC training drill compared to controls. However, training did not improve autonomic stress responses, and the authors did not report post-training performance results (Page et al., 2016). While these findings suggest a modest added benefit of mental skills training to motor learning paradigms, direct comparison of different learning strategies have shown that combined physical and mental practice is not as effective in training procedural skills compared to 100% physical practice (Hird et al., 1991).

#### Live Versus Virtual Scenario-Based Training

Despite the efficacy of the training interventions by Oudejans and colleagues discussed in section “The Adaptive Role of Real-World Stress in Police Training,” the test and training conditions were not truly scenario based, such that officers were instructed to shoot at targets (or individuals) that were directly in front of them, and no other suspect engagement (i.e., verbal communication, other physical tactics) or decision-making (i.e., deciding whether or not to shoot the target) was involved. It is also unclear what duration of training, experimentally induced practice effects, or occupational experience could result in performance improvements, as the low-stress training group performed equally well during the high-stress condition at 4-month follow-up (Nieuwenhuys and Oudejans, 2011). One investigation also found no post-training improvements in performance or gaze control, which could be due to the use of a video presentation for the stimuli instead of live actors (Nieuwenhuys et al., 2015).

There is increasing investment in virtual simulation technologies for occupational training among police agencies and training institutes. However, there has not been any empirical validation of simulators for police training, specifically in the use of force decision-making. One validation study found that virtual emergency medical training failed to induce the same level of cardiovascular stress as a live training scenario (Baker et al., 2017). Despite the appeal of using advanced technologies in an applied setting, video or virtual simulators lack the perceptual depth cues present in live environments that inform police decision-making and motor selection strategies. Further empirical validation of simulator systems relative to live scenario-based training is needed before police agencies make the considerable investment in implementing these methods for the use of force training.

#### Future Directions for Evidence-Based Police Training: Neurophysiological Mechanisms

While the studies reviewed so far have helped reveal the peripheral cardiovascular physiological mechanisms of police performance under stress, investigations are lacking on the impact of stress on central neurological mechanisms underlying police behavior. Stress has typically been operationalized with measures of HR, HRV, and cortisol, but neurological patterns measured by EEG scalp electrodes can also indicate increased anxiety or threat. Asymmetry in the extent of activation in left versus right frontal cortex is related to emotional and motivational processing (for review, see Harmon-Jones et al., 2010) and may predict which officers are more (or less) susceptible to stress-induced impairments in perception or performance. In a single pilot study, Johnson et al. (2014) investigated the differences in psychophysiological measures of HR, HRV, EEG, and lethal force decision-making (i.e., correct responses and errors) between civilians and military or police experts during high-stakes video scenarios. In addition to significantly higher pass rates, researchers found expertise-driven differences in HR and EEG measures but not HRV or alpha asymmetry. Further, these effects were greater for experts with more experience (10+ years versus intermediate experts with 6–10 years of experience). In spite of the study’s limitations, this preliminary investigation is an important step in the right direction toward understanding the complex relationship between training/experience and different biological systems to high-stakes decision-making by police.

Other brain signals that can shed light on the neural correlates of police learning and behavior include the error-related negativity (ERN), which occurs within milliseconds following motor execution to monitor action and detect errors, as with reafferent feedback during motor learning (see section “Reinforcing Motor Learning in the Brain”). ERNs are enhanced among highly anxious individuals (Hajcak et al., 2003) and are sensitive to internal appraisals of threat (Weinberg et al., 2016). Enhanced ERNs were also observed following false positive identification of tools as weapons by military cadets primed by a threatening stimulus (Fleming et al., 2010), establishing a clear link between brain signals preceding or generated by the use of force decisions (both correct and incorrect) under stressful conditions.

A single pilot study on athletes compared the effects of HRV-BF training on cardiovascular and neurophysiological measures of arousal but not physical performance. For the HRV-BF group only, results show reductions in reported anxiety, increased HRV amplitude indicative of increased vagal tone and enhanced parasympathetic activity, as well as reduced frontal asymmetry and improved emotional control (Dziembowska et al., 2016). Future studies investigating the efficacy of HRV-BF training on police including iPREP can perform similar analyses on the bidirectional communication between central neurological and peripheral physiological systems and compare these biological markers to objective performance measures.

## Clinical Applications of Complex Motor Learning

The neurophysiological mechanisms underlying motor learning in a law enforcement context have been summarized above from the lens of cognitive neuroscience, with a call for more applied research that investigates police in occupationally relevant settings outside of a laboratory. Further insights on how motor learning is facilitated and stored in the brain are provided by examining clinical populations that experience breakdowns in these mechanisms, including people with Alzheimer’s disease (AD), Parkinson’s disease (PD), stroke, and traumatic brain injuries. This review will not go into detail on the prevalence of, and therapies for, psychological injuries and mental health disorders common among law enforcement (e.g., Carleton et al., 2018) but rather will consider the brain-based therapeutic benefits of complex motor learning.

### Motor Learning Mechanisms Revealed by Disease-Related Impairments

Several clinical populations have demonstrated various deficits in how they chunk or segment continuous streams of movement. These deficits result in significant impairments to the “online” or real-time perception, and subsequent learning and memory, of motor information. Research on individuals with PD (Tremblay et al., 2010) and stroke patients (Boyd et al., 2009) show impaired concatenation of motor chunks, suggesting a crucial role for the basal ganglia in understanding and consolidating movement sequences into long-term memory (Yin and Knowlton, 2006). Other clinical populations, including patients with schizophrenia (Zalla et al., 2004) and frontal lobe lesions (Zalla et al., 2003), show deficits in segmentation ability such that the location of event borders vary from normative ones. Individuals with mild dementia and AD also show poor recognition and order memory of segmented action (Zacks et al., 2006), demonstrating a clear link between online attention, visuomotor, and memory functioning.

Visuomotor deficits have been revealed in clinical populations using simple tasks (e.g., moving a cursor on a screen with one’s finger) presented on touchscreen tablets that can record reaction times and movement accuracy as proxy measurements of motor recalibration and adaptation (section “Reinforcing Motor Learning in the Brain,” e.g., Tippett and Sergio, 2006). Not only is this methodology useful in determining disease-related impairments in visuomotor functioning (for review, see Joddrell and Astell, 2016), modified reaction time and adaptation tasks could also be used as screening and/or training tools for policing skills similar to their application in athletics as performance and injury evaluation tools (Ventura et al., 2016).

### Therapeutic Benefits of Complex Motor Learning

Based on the neurophysiological mechanisms underlying complex motor learning described above, the following section will review the therapeutic application of complex motor learning for movement and memory disorders. There is demonstrated efficacy for improved attention and memory following relatively short (18 min) training with simple eye movements (Di Noto et al., 2013), including eye movement desensitization and reprocessing (EMDR) therapy. While EMDR has shown significant neurological and clinical improvements in post-traumatic stress disorder (PTSD) among police officers involved in on-duty shootings (Lansing et al., 2005), the following sections go beyond traditional forms of simple movement therapy (i.e., physical, pharmacological) to review how multisensory dance and music practice facilitate perception, understanding, and learning of complex sequences of movement.

Various forms of music and dance practice have been employed as alternative forms of therapy for a wide variety of disorders (for comprehensive review of therapies and specific outcomes, see Dhami et al., 2015). Especially during the last 25 years, music therapy has become internationally recognized as part of health care maintenance and rehabilitation, with systematic developments in training and research during this time (Ala-Ruona, 2007). In addition to improving primary disease symptoms and increased functional connectivity between motor planning (SMA) and control (cerebellum) regions of the brain, music and dance therapy have proven social and emotional benefits, including improved reports of mood, anxiety, and quality of life among individuals with AD-related dementia and PD (Heiberger et al., 2011; Westheimer et al., 2015; King et al., 2019). The holistic benefits of dance and music therapy for general well-being are significant, as an estimated 35% of individuals with PD also exhibit symptoms of depression (Reijnders et al., 2008). Further, the social and emotional benefits of dance and music therapy are suggested to drive high rates of adherence, which in turn contributes to the efficacy of these types of therapy for individuals suffering from impaired motor and memory functioning.

#### Seeing and Hearing Is Believing: Superadditive Mechanisms of Multisensory Inputs

By their nature, dance and music engage multimodal (i.e., sensory, motor, and memory) regions of the brain that individually may be affected by disease or injury. Regular participation in dance or music therapy capitalizes on the neurophysiological mechanisms underlying complex motor learning to recover functioning in one of two ways: by promoting neural activation in damaged multimodal brain regions and/or by forging novel and alternate neural pathways among multimodal networks. Similar to Hebbian principles described in section “Reinforcing Motor Learning in the Brain,” individuals with AD show impaired STDP between regions of the MNN (Figure 1). reflecting impaired learning mechanisms (Di Lorenzo et al., 2018). Just as research in healthy individuals reveal how the brain retains new information, investigations on clinical populations can demonstrate how neurological processes manifest in functional (i.e., cognitive, motor) impairments.

Therapeutic efficacy of music and dance are also potentially driven by additive (or superadditive) activation of multisensory neurons that fire equal to (or greater) than the sum of two unisensory inputs (Stevenson et al., 2007; Werner and Noppeney, 2010). That is, concurrent presentation of visual and auditory stimuli will elicit greater activation from the same neurons than when either stimulus is presented on its own. Just as in fundamental motor learning, the neural representations of novel dance and music-producing movements are reinforced by reafferent feedback. However, music and dance movements are additively reinforced by external musical (i.e., auditory) stimuli together with reafferent feedback signals from multiple sensory inputs, including vision, audition, and proprioception (sense of body position and movement). When there is a failure to distinguish self-generated efference copy signals from external sensory stimuli, it can lead to perceived hallucinations as seen in schizophrenia (Pynn and DeSouza, 2013). Multisensory cues are also present in and relevant to police contexts, especially for situational awareness (see section “Situational Awareness”) and should be integrated into procedural training to reinforce motor learning.

#### Entrainment of External and Internal Rhythms to Promote Learning and Recover Disease-Related Functioning

Music and dance typically involve regular rhythmic patterns, which are also present in various physiological functions including heart rate, respiration, and gait (i.e., walking). The activation of billions of neurons also produces oscillatory brain rhythms of various frequencies, much like the different frequencies of radio stations. Internally generated biological rhythms can be paired to, or cued by, external rhythms by a mechanism known as “entrainment” (for review, see Thaut, 2015). Driven by basic principles in physics, entrainment causes two asynchronous frequencies to coordinate themselves into a common or synchronous period. A stronger or faster frequency, such as that provided by an external stimulus (e.g., metronome, musical beat), will lock a weaker or slower frequency (e.g., neural activity) into a stable rhythmic period.

A growing body of evidence on dance therapy for people with PD has shown almost immediate (i.e., after a single session) and lasting improvements in disease-related impairments to gait, rigidity, balance, and tremor (Heiberger et al., 2011; Westheimer et al., 2015; Bearss et al., 2017). Improvements in motor functioning following dance therapy are suggested to be mediated by entrainment mechanisms (Thaut et al., 1996; McIntosh et al., 1997), whereby dominant external musical rhythms offset the slowing of brain rhythms observed in people with PD (Soikkeli et al., 1991; Moazami-Goudarzi et al., 2008). These findings bear significant implications for the application of dance and music therapy for police officers and other individuals with PTSD, which has shown a breakdown in cross-frequency communication between emotional and sensory-motor brain regions (Cohen et al., 2013). By repairing neurophysiological rhythms through training with external rhythmic stimuli, research shows that improvements in motor functioning can potentially translate to improvements in emotional processing as well, reducing the lasting negative impact of disease, injury, and trauma.

Together, these findings synthesize seminal research from basic and clinical neuroscience to illuminate how learning-induced neuroplasticity facilitates the recovery of motor and cognitive functioning for various clinical disorders. Identifying the neurophysiological mechanisms underlying effective movement therapies provides significant insights for the development of police training programs that can withstand the realities of occupationally relevant situations.

## Challenges to Implementing Evidence-Based Police Practices

Given the wealth of multidisciplinary empirical evidence presented thus far on complex motor learning as relevant to policing, there remain several practical, organizational, and systemic challenges to implementing evidence-based approaches. We will briefly review some of these challenges before providing recommendations for best practices in police training.

### How Much Training Is Needed? Establishing Universal Standards

#### Basic Recruit Training Versus Continued Education at the Agency Level

A problem that exists in the practical application of motor learning research is the specification of precisely how many repetitions or hours of training are required to develop adequate competency. Individual differences in physical, cognitive, and learning abilities further complicate identifying a universally prescribed training regimen. This problem is especially relevant for policy makers and educators in all industries, who are challenged to balance finite resources with maintaining occupational safety and performance standards that are often highly variable and poorly defined.

Police training begins with an introduction to the basic skills that an officer will require and use in their day-to-day practice, which is subsequently expanded upon and specialized at the local agency or precinct level. There is no universal occupational standard for the duration or content of basic (or extended) police training and varies across jurisdictions. For instance, the Justice Institute of British Columbia (JIBC) delivers basic recruit training for 12 police agencies in Western Canada post-hire. In addition to a minimum requirement of 2 years of post-secondary education, officers partake in a 38-week basic training program that includes a middle block of field within their employers’ police agency. In contrast, all police officers in Finland complete a 3-year Bachelor of Police Services at the national Police University College (PUC), qualifying them for full-time employment at any local precinct in the country. Trainees complete approximately 40 weeks of mandatory in-field training to develop professional competencies during the 3-year period. Reviewing and contrasting current training programs lie beyond the scope of this review (see also section “Cultural Challenges and Access to Information” regarding access to this information), but the two basic recruit programs described above reflect relatively long training durations. Relative to other occupations that require high-stakes life-or-death decision-making (e.g., surgeons, emergency medical personnel) and several years of basic and specialized training, even the JIBC and PUC programs are significantly shorter. Given the complexity and inherent stress associated with police work, training should adequately prepare officers from all backgrounds to safely meet occupational demands.

#### Learning Theory and the “10,000 h” Principle

One of the earliest theories quantifying the progression of motor learning is Galton’s (1869) *lifespan development theory*, which states that this ceiling in performance is bound by immutable biological genetic factors including physical (i.e., height, weight, body composition) and mental attributes (i.e., intelligence). More recently adapted by Fitts and Posner (1967), it is suggested that expert-level performance can be reached with approximately 50 h of training, after which no additional amount of training will further improve an individual’s performance. However, these training paradigms evaluated simplistic physical skills. As such, 50 h of basic firearms training may result in expert performance of this skill but is likely not sufficient for more complex physical and mental skills (i.e., deciding how and when to use a firearm, or any other tactical option, during a variety of stressful encounters).

Yet another estimate of prescribed training time to achieve expert-level skill is based on Simon and Chase’s (1973) investigations of elite chess players, whose successful performance at international competitive tournaments was not possible without at least a decade of practice. These findings were popularized to form the “10,000 h rule” (Gladwell, 2008), but Simon and Chase (1973) posited that expertise arises from repeated exposure to an increased variety of chess combinations and strategies. Each of these unique scenarios created stored memories for successful or failed experiences that experts could rely on to promote future success in similar situations. Similar to the principles of intuitive decision-making described earlier, scenario-based training exposes officers to multiple varied encounters that can inform the best course of action in similar future situations. Many more hours of training and occupational experience are needed to develop a repertoire of high-level skills (e.g., use of force decision-making, situational awareness) that can be flexibly and accurately applied to a variety of circumstances, relative to basic skill competency.

#### Tracking Motor Learning in the Brain Through Neuroplasticity

Neuroscientists have tried to observe incremental changes to brain structure and function following novel motor skill learning. Observable increases in structural gray matter (i.e., neural cell bodies and synaptic connections between them) are evident as early as 1 or 2 weeks of daily practice, and decaying 2–4 months once training has stopped (Driemeyer et al., 2008). However, functional neuroimaging research suggests learning-induced plasticity or reorganization of synaptic connections in the brain as early as the very first training session. Karni et al. (1995) identified a switch between initial “fast learning” and consolidated “slow learning” that corresponds with improvements in performance speed and accuracy and less variability across movement trials (Shmuelof et al., 2012). Further, M1 activity increased over several weeks of daily practice and was maintained for up to 5 months with no additional training (Karni et al., 1995). Cross et al. (2009) conducted a neuroimaging study comparing physical and observational training of brief dance sequences among non-experts. After only 5 days of training, they found common gains in activation of premotor and inferior parietal MNN regions (Figure 1) but better performance and significant M1 activation for physically versus visually trained dance sequences (Cross et al., 2009).

Lending empirical evidence to the principle of “use it or lose it,” these findings show fast initial training gains that decay relatively quickly once training ends. Although they did not measure neurological indexes of motor learning, Andersen et al. (2018) investigated skill decay following a 4-day iPREP training program. Officers’ gains in performance (reduction of lethal force errors) and autonomic stress regulation (decreased maximum HR and faster recovery to resting HR) were maintained up to 12-month post-training, returning to pre-training levels at 18-month follow-up. Together with the neuroscientific evidence presented here, these findings underscore the importance of continued practice and regular refresher training to maintain learning-induced changes to performance, brain structure, and function.

### Organizational Challenges

#### Leveraging Finite Training Resources: Funding and Qualified Personnel

Before adopting evidence-based training approaches, individual police agencies must leverage finite available resources, including time away from regular duties, funding, and qualified personnel. Scenario-based training is especially resource-intensive and educationally challenging, despite demonstrated efficacy. Many unique and relevant scenarios need to be developed and executed with a high degree of realism to preserve the value of the teaching opportunity. However, evidence-based training may facilitate cost savings in the long run. In an evaluation commissioned by the United States Department of Education of 77 educational interventions that were not evidence-based, 91% were found to have weak or no positive effects (Coalition for Evidence-Based Policy, 2014). The United States military also implemented a $125 million dollar program (Comprehensive Soldier Fitness) to enhance resilience and performance before evaluating its efficacy, which was found to have no objective improvements and at worst may actually cause harm (McCord, 2003; Eidelson et al., 2011). Krameddine et al. (2013) performed a cost-benefit analysis of their scenario-based mental health training program and found that savings incurred from a significant reduction in time spent on mental health calls exceeded the cost of the study and training program. These outcomes are especially significant given a higher incidence of subsequent mental health calls, further underscoring the compelling nature of an evidence-based approach to police training.

In addition to training content and method of delivery, trainers’ skills, abilities, and approach are very important from both educational (i.e., during training) and professional (i.e., during regular police work) perspectives (Murphy, 2014), yet no standards or definitions exist for proficient trainers. Novel pedagogical approaches in policing have been explored, including an emphasis on group discussion and active debate (Birzer, 2003) and the development of “train the trainer” programs (Hammerness et al., 2005, 2007; Darling-Hammond and Bransford, 2007). Qualified trainers are often “those that do” and may not have formal pedagogical training to identify or address the unique learning needs of their trainees. To address this gap in occupational training, Finland’s PUC has introduced a 6-week teaching course specifically designed for use of force trainers. In addition to subject knowledge, the course covers general pedagogy including recognizing and solving challenges of the training group. Other learning outcomes include independently organizing a training event and training key issues related to the selection and use of force in a pragmatic and fundamental manner. In addition to a lack of empirical data on police training outcomes, there is a scarcity of research or data on teaching effectiveness. This has led to the definition of training objectives and quality criteria according to available resources over practical consideration of the skills intended for training. Therefore, the systematic evaluation and development of police pedagogy is limited and vulnerable to approaches that are not evidence-based, compromising the learning opportunity for trainees.

#### Cultural Challenges and Access to Information

There are strong differences in opinion surrounding police training and practices more general among various stakeholders, including policy makers, police supervisors, trainers, management, officers, as well as the public. Due to the sensitive nature of training content (i.e., specific tactical plans and maneuvers that involve lethal force), information is often kept secret from the public to maintain safety. However, the confidential nature of police training practices can also foster a culture of unwillingness to share ideas among stakeholders and creates an additional barrier to collecting and comparing useful information to promote best practices. Updating existing police training models is also met with a great deal of controversy, despite mounting empirical evidence for the benefits of various training approaches including problem-based learning (Makin, 2016) and a societal need for changes to various policing practices. However, newer motor learning models currently used in military training have adopted a more holistic approach and consider factors like environment, cognitive skills, and kinematic movement options on various continuums (Schmidt et al., 2019). The primary goal of effective training for stressful and unpredictable situations is to protect both public and police and should not be compromised due to extraneous factors (i.e., resources, personal, or political motivations).

Similarly, academic research is often inaccessible to police practitioners unless findings are published in open-access format at a significant financial cost to the researchers. This “silo effect” of information stunts knowledge exchange between sectors and is a barrier to disseminating useful research evidence to practical end-users. Despite containing very relevant insights for policing, scientific articles laden with jargon and field-specific terminology are often not translated into a language that is accessible or understandable to an applied audience or the general public. However, there are peer-reviewed journals and publications available that seek to combine academic and applied perspectives in policing, including *Police Practice and Research*, *Policing and Society*, and *Policing: An International Journal.* Evidence-based reports are also published in industry-specific periodicals such as *The Police Chief* and *The Blue Line*. Indeed, the purpose of this Special Edition of *Frontiers* is to disseminate relevant and timely knowledge across domains for the purpose of improving current practices in policing, identifying areas of future research and development, and saving time and resources by understanding what has already been done.

## Recommendations: Best Practices for Evidence-Based Police Training

Common practice should not be confused with best practice or evidence-based practice. The following recommendations are based on the empirical and applied research evidence summarized in this review and are intended to promote training effectiveness as well as occupational safety.

### Training

1. Officers need to be prepared for the perceptual and physiological impacts of stress that they will experience on the job (section “The Influence of Stress on Police Performance”). An important consideration for developers of police training programs is to identify an optimal level of stress that adaptively promotes learning without crossing the threshold for maladaptive stress that interferes with encoding and retrieval processes (Figure 2).
2. In addition to the benefits outlined in section “The Gold Standard for Complex Motor Learning for Police: Scenario-Based Training,” scenario-based training facilitates motor learning by inducing realistic levels of occupational stress that helps override perceptual distortions and preferred movement strategies that could put the officer at risk. In addition, the physiological arousal elicited by scenario-based training can promote adherence to and active engagement with training, maximizing the learning opportunity afforded during limited training time with finite resources.
3. Police work is similar around the world; regardless of the laws and regulations specific to their jurisdictions, officers are uniquely tasked with addressing the needs of people in crisis. Without investigation, standardization, and validation of training strategies, police officers may be learning wrong or ineffective patterns, and encoding stimulus-response tendencies instead of effective critical thinking skills. On a larger scale, standardization of minimal training requirements should align practices across jurisdictions, as is currently done in Finland and under development in Canada (Canadian Association of Chiefs of Police, 2018).
4. Training delivery (i.e., duration, methods) needs to be appropriate to the skills intended to be trained. As such, complex motor, verbal, and cognitive skills including situational awareness, decision-making, and de-escalation should be trained in live environments with trained actors or instructors that can dynamically respond to officers’ behaviors. Further empirical validation of virtual simulator systems relative to live scenario-based training is needed before police agencies make the considerable investment in implementing these methods for the use of force training. However, virtual technology can be useful as complimentary training tools (i.e., in addition to contextually relevant training settings) as well as for psychophysical performance screening similar to its application in athletics (section “Live Versus Virtual Scenario-Based Training”).
5. Action competence and an officer’s self-confidence should be considered by trainers and curriculum developers when reimagining police training methods and approaches (section “Confidence and Action Competency”).
6. Through more introspective pedagogical approaches, police trainers can use mistakes in both novice and expert officers’ performance to recalibrate and reinforce correct intuitive motor and cognitive strategies when training situational awareness and use of force decision-making.
7. By repairing neurophysiological rhythms through training with external rhythmic stimuli, clinical research shows that improvements in motor functioning can potentially translate to improvements in emotional processing as well (section “Clinical Applications of Complex Motor Learning”), reducing the lasting negative impact of disease, injury, and trauma. Officers are encouraged to seek out extracurricular activities that incorporate social engagement and rhythmic and/or multisensory components such as music, dance, or athletics to promote physical and emotional health.

### Knowledge Dissemination

8. To facilitate knowledge exchange between academic and applied professionals, and police practitioners around the world and across jurisdictions, new research should be published in open-access journals, and practitioners should attend relevant conferences and workshops wherever possible, and when resources permit. Becoming involved and engaged with research will not only help generate new knowledge but will provide police practitioners with an understanding of the scientific process, from generating a research question, to implementing an experimental study, and observing and communicating the results.
9. Police management should recognize and accept the importance of evidence-based policing and offer their trainers and officers opportunities to access and/or engage in applied research with partnerships at local academic institutions.
10. More importantly, a systematic cultural change within policing needs to occur in which stakeholders from multiple levels and sectors can meet and openly share knowledge and be willing to accept evidence in favor of opinions fuelled by political, financial, or personal motives.

## Conclusions

An elite athlete such as a javelin thrower has one precise task to perform under a narrow range of controlled conditions. In police work, even a simple motor skill is never standardized or used in isolation. Officers have to constantly evaluate, consider, decide, and update what is appropriate or possible given the unfolding situation. As such, motor skills cannot be considered without all other aspects of police encounters, including occupationally relevant stress, situational awareness, and complex decision-making.

The current review is a synthesis of empirical and applied research on the fundamental principles of motor learning as relevant to police. Insights from the fields of applied policing, cognitive and computational neuroscience, and clinical and health psychology lend empirical evidence to the knowledge inherently possessed by police trainers, officers, and practitioners through their first-hand experience. Especially relevant to law enforcement, we consider the influence of occupationally relevant stress on the physiological and neurological mechanisms underlying police learning and performance. Training policies and protocols should be updated accordingly to reflect current knowledge and to promote motor learning and skill retention.

Bridging research across fields and industries also provides solutions to several systemic challenges to evidence-based policing, including a lack of universal training standards and knowledge exchange. Ultimately, we hope that this review will inspire practitioner engagement with applied research, and spark open and productive debate among various stakeholders on best practices surrounding training the complex motor skills required in policing.

## Author Contributions

PD and J-MH wrote the manuscript, conducted all background literature research to inform the contents, and approve the submitted version.

### Conflict of Interest Statement

The authors declare that the research was conducted in the absence of any commercial or financial relationships that could be construed as a potential conflict of interest.

## Footnotes

^1^Some of the noted benefits of visualization (or mental imagery, motor imagery, or mental simulation) include the rehearsal of visual, motor/kinesthetic, spatial, and symbolic aspects of a given movement in the absence of overt movement (Sherwood and Lee, 2003). Visualization as a proposed component of intuitive decision-making and an effective training tool in the context of policing are discussed below.

## References

[ref1] Akinola M. Mendes W. B. (2012). Stress-induced cortisol facilitates threat-related decision making among police officers. Behav. Neurosci. 126, 167–174. 10.1037/a0026657, PMID: 22141468

[ref2] Ala-Ruona E. (2007). Initial assessment as a clinical procedure in music therapy of clients with mental health problems - strategies, methods and tools (Dissertation). Jyväskylä Stud. Humanit. 91 (ISSN 1459-4331).

[ref3] Aldred K. (2017). Be confident, practise skills, stay healthy: Four RCMP instructors talk training. RCMP Gazette, 79. Available at: http://www.rcmp-grc.gc.ca/en/gazette/be-confident-practise-skills-stay-healthy (Accessed December 3 2018).

[ref4] Andersen J. P. Di Nota P. M. Beston B. Boychuk E. C. Gustafsberg H. Poplawski S.. (2018). Reducing lethal force errors by modulating police physiology. J. Occup. Environ. Med. 60, 867–874. 10.1097/JOM.0000000000001401, PMID: 30020222 PMC6200377

[ref5] Andersen J. P. Gustafsberg H. (2016b). A training method to improve police use of force decision making: a randomized controlled trial. SAGE Open 6, 1–13. 10.1177/2158244016638708

[ref6] Andersen J. P. Pitel M. Weerasinghe A. Papazoglou K. (2016a). Highly realistic scenario based training simulates the psychophysiology of real world use of force encounters: implications for improved police officer performance. J. Law Enforcement 5, 1–13. http://hdl.handle.net/1807/73822

[ref7] Anderson G. S. Litzenberger R. Plecas D. (2002). Physical evidence of police officer stress. Policing 25, 399–420. 10.1108/13639510210429437

[ref8] Armstrong J. Clare J. Plecas D. (2014). Monitoring the impact of scenario-based use-of-force simulations on police heart rate: evaluating the Royal Canadian Mounted Police Skills Refresher Program. Criminology, Crim. Just. Law Soc. 15, 51–59. http://wcr.sonoma.edu/v15n1/Armstrong.pdf

[ref9] Arnetz B. B. Nevedal D. C. Lumley M. A. Backman L. Lublin A. (2009). Trauma resilience training for police: psychophysiological and performance effects. J. Police Crim. Psychol. 24, 1–9. 10.1007/s11896-008-9030-y

[ref10] Assaf Y. Pasternak O. (2007). Diffusion tensor imaging (DTI)-based white matter mapping in brain research: a review. J. Mol. Neurosci. 34, 51–61. 10.1007/s12031-007-0029-0 18157658

[ref11] Baker B. G. Bhalla A. Doleman B. Yarnold E. Simons S. Lund J. N.. (2017). Simulation fails to replicate stress in trainees performing a technical procedure in the clinical environment. Med. Teach. 39, 53–57. 10.1080/0142159X.2016.1230188, PMID: 27631579

[ref12] Bar R. J. DeSouza J. F. X. (2016). Tracking plasticity: effects of long-term rehearsal in expert dancers encoding music to movement. PLoS One 11:e0147731. 10.1371/journal.pone.0147731, PMID: 26824475 PMC4732757

[ref13] Barney C. Shea S. C. (2007). The art of effectively teaching clinical interviewing skills using role-playing: a primer. Psychiatr. Clin. N. Am. 30, 31–50. 10.1016/j.psc.2007.03.001 17643829

[ref14] Barrett L. F. (2012). Emotions are real. Emotion 12, 413–429. 10.1037/a0027555, PMID: 22642358

[ref15] Barrett L. F. Bar M. (2009). See it with feeling: affective predictions during object perception. Philos. Trans. R. Soc. Lond. B Biol. Sci. 364, 1325–1334. 10.1098/rstb.2008.0312, PMID: 19528014 PMC2666711

[ref16] Baumann J. DeSteno D. (2010). Emotion guided threat detection: expecting guns where there are none. J. Pers. Soc. Psychol. 99, 595–610. 10.1037/a0020665, PMID: 20731499

[ref17] Bearss K. A. McDonald K. C. Bar R. J. DeSouza J. F. (2017). Improvements in balance and gait speed after a 12 week dance intervention for Parkinson’s disease. Adv. Integr. Med. 4, 10–13. 10.1016/j.aimed.2017.02.002

[ref18] Binkofski F. Buccino G. (2006). The role of ventral premotor cortex in action execution and action understanding. J. Physiol. Paris 99, 396–405. 10.1016/j.jphysparis.2006.03.005, PMID: 16723210

[ref19] Birzer M. L. (2003). The theory of andragogy applied to police training. Policing 26, 29–42. 10.1108/13639510310460288

[ref503] Blair J. P. Pollock J. Montague D. Nichols T. Curnutt J. Burns D. (2011). Reasonableness and reaction time. Police Q. 14, 323–343. 10.1177/1098611111423737, PMID: 26459346

[ref20] Blakemore S.-J. Rees G. Frith C. D. (1998). How do we predict the consequences of our actions? A functional imaging study. Neuropsychologia 36, 521–529. 10.1016/S0028-3932(97)00145-0, PMID: 9705062

[ref21] Bläsing B. E. (2015). Segmentation of dance movement: effects of expertise, visual familiarity, motor experience and music. Front. Psychol. 5, 353–311. 10.3389/fpsyg.2014.01500 PMC4285866 25610409

[ref22] Bo J. Seidler R. D. (2009). Visuospatial working memory capacity predicts the organization of acquired explicit motor sequences. J. Neurophysiol. 101, 3116–3125. 10.1152/jn.00006.2009, PMID: 19357338 PMC2694099

[ref23] Boyd L. A. Edwards J. D. Siengsukon C. S. Vidoni E. D. Wessel B. D. Linsdell M. A. (2009). Motor sequence chunking is impaired by basal ganglia stroke. Neurobiol. Learn. Mem. 92, 35–44. 10.1016/j.nlm.2009.02.009, PMID: 19249378

[ref24] Cahill L. Alkire M. T. (2003). Epinephrine enhancement of human memory consolidation: interaction with arousal at encoding. Neurobiol. Learn. Mem. 79, 194–198. 10.1016/S1074-7427(02)00036-9, PMID: 12591227

[ref25] Cahill L. McGaugh J. L. (1998). Mechanisms of emotional arousal and lasting declarative memory. Trends Neurosci. 21, 294–299. 10.1016/S0166-2236(97)01214-9, PMID: 9683321

[ref26] Canadian Association of Chiefs of Police (2018). Human resources & learning committee annual report 2017–2018.

[ref27] Carleton R. N. Afifi T. O. Taillieu T. Turner S. Krakauer R. Anderson G. S. (2019). Exposures to potentially traumatic events among public safety personnel in Canada. Can. J. Behav. Sci. 51, 37–52. 10.1037/cbs0000115

[ref28] Carleton R. N. Afifi T. O. Turner S. Taillieu T. Duranceau S. LeBouthillier D. M. (2018). Mental disorder symptoms among public safety personnel in Canada. Can. J. Psychiatry 63, 54–64. 10.1177/0706743717723825 28845686 PMC5788123

[ref29] Cisek P. (2006). Integrated neural processes for defining potential actions and deciding between them: a computational model. J. Neurosci. 26, 9761–9770. 10.1523/JNEUROSCI.5605-05.2006, PMID: 16988047 PMC6674435

[ref30] Cisek P. (2007). Cortical mechanisms of action selection: the affordance competition hypothesis. Philos. Trans. R. Soc. Lond. B Biol. Sci. 362, 1585–1599. 10.1098/rstb.2007.2054, PMID: 17428779 PMC2440773

[ref31] Cisek P. Kalaska J. F. (2004). Neural correlates of mental rehearsal in dorsal premotor cortex. Nature 431, 993–996. 10.1038/nature03005, PMID: 15496925

[ref32] Coalition for Evidence-Based Policy (2014). Which study designs are capable of producing valid evidence about a program’s effectiveness? Available at: http://coalition4evidence.org/wp-content/uploads/2014/10/Which-Study-Designs-areCapable-of-Producing-Valid-Evidence-of-Effectiveness.pdf (Accessed May 15 2017).

[ref33] Cohen J. E. Shalev H. Admon R. Hefetz S. Gasho C. J. Shachar L. J.. (2013). Emotional brain rhythms and their impairment in post-traumatic patients. Hum. Brain Mapp. 34, 1344–1356. 10.1002/hbm.21516, PMID: 22331598 PMC6870431

[ref34] Cressman E. K. Henriques D. Y. P. (2009). Sensory recalibration of hand position following visuomotor adaptation. J. Neurophysiol. 102, 3505–3518. 10.1152/jn.00514.2009, PMID: 19828727

[ref35] Cross E. S. Kraemer D. J. M. Hamilton A. F. D. C. Kelley W. M. Grafton S. T. (2009). Sensitivity of the action observation network to physical and observational learning. Cereb. Cortex 19, 315–326. 10.1093/cercor/bhn083, PMID: 18515297 PMC2638791

[ref36] Darling-Hammond L. Bransford J. (2007). Preparing teachers for a changing world: What teachers should learn and be able to do. Hoboken, NJ: John Wiley & Sons.

[ref37] De Quervain D. J. Roozendaal B. McGaugh J. L. (1998). Stress and glucocorticoids impair retrieval of long-term spatial memory. Nature 394, 787–790. 10.1038/29542, PMID: 9723618

[ref38] De Quervain D. J. Roozendaal B. Nitsch R. M. McGaugh J. L. Hock C. (2000). Acute cortisone administration impairs retrieval of long-term declarative memory in humans. Nat. Neurosci. 3, 313–314. 10.1038/73873, PMID: 10725918

[ref39] DeSouza J. F. X. Menon R. S. Everling S. (2003). Preparatory set associated with pro-saccades and anti-saccades in humans investigated with event-related fMRI. J. Neurophysiol. 89, 1016–1023. 10.1152/jn.00562.2002, PMID: 12574477

[ref40] Dhami P. Moreno S. DeSouza J. F. (2015). New framework for rehabilitation–fusion of cognitive and physical rehabilitation: the hope for dancing. Front. Psychol. 5:1478. 10.3389/fpsyg.2014.01478 25674066 PMC4309167

[ref41] Di Lorenzo F. Ponzo V. Motta C. Bonnì S. Picazio S. Caltagirone C.. (2018). Impaired spike timing dependent cortico-cortical plasticity in Alzheimer’s disease patients. J. Alzheimers Dis. 66, 983–991. 10.3233/JAD-180503, PMID: 30372679

[ref42] Di Nota P. M. (2017). Short-and long-term changes in attention, memory and brain activity following exercise, motor learning, and expertise. Dissertation. Toronto, (ON): York University.

[ref43] Di Noto P. M. Uta S. DeSouza J. F. X. (2013). Eye exercises enhance accuracy and letter recognition, but not reaction time, in a modified rapid serial visual presentation task. PLoS One 8:E59244. 10.1371/journal.pone.0059244, PMID: 23527146 PMC3602039

[ref44] Diamond D. M. Campbell A. M. Park C. R. Woodson J. C. Conrad C. D. Bachstetter A. D.. (2006). Influence of predator stress on the consolidation versus retrieval of long-term spatial memory and hippocampal spinogenesis. Hippocampus 16, 571–576. 10.1002/hipo.20188, PMID: 16741974

[ref45] Doane S. M. Pellegrino J. W. Klatzky R. L. (1990). Expertise in a computer operating system: conceptualization and performance. Hum. Comput. Interact. 5, 267–304. 10.1207/s15327051hci0502&3_5

[ref46] Driemeyer J. Boyke J. Gaser C. Büchel C. May A. (2008). Changes in gray matter induced by learning—revisited. PLoS One 3:e2669–5. 10.1371/journal.pone.0002669, PMID: 18648501 PMC2447176

[ref47] Dziembowska I. Izdebski P. Rasmus A. Brudny J. Grzelczak M. Cysewski P. (2016). Effects of heart rate variability biofeedback on EEG alpha asymmetry and anxiety symptoms in male athletes: a pilot study. Appl. Psychophysiol. Biofeedback 41, 141–150. 10.1007/s10484-015-9319-4, PMID: 26459346

[ref48] Eidelson R. Pilisuk M. Soldz S. (2011). The dark side of comprehensive soldier fitness. Am. Psychol. 66, 643–644. 10.1037/a0025272, PMID: 21967209

[ref49] Endsley M. R. (1995). Toward a theory of situation awareness in dynamic systems. Hum. Factors 37, 32–64. 10.1518/001872095779049543

[ref50] Ericsson K. A. (1998). The scientific study of expert levels of performance: general implications for optimal learning and creativity. High Abil. Stud. 9, 75–100. 10.1080/1359813980090106

[ref51] Ericsson K. A. (2004). Deliberate practice and the acquisition and maintenance of expert performance in medicine and related domains. Acad. Med. 79, S70–S81. 10.1097/00001888-200410001-00022 15383395

[ref52] Ericsson K. A. Chase W. G. Faloon S. (1980). Acquisition of a memory skill. Science 208, 1181–1182. 10.1126/science.7375930 7375930

[ref53] Ericsson K. A. Krampe R. T. Tesch-Römer C. (1993). The role of deliberate practice in the acquisition of expert performance. Psychol. Rev. 100, 363–406. 10.1037/0033-295X.100.3.363

[ref54] Ericsson K. A. Lehmann A. C. (1996). Expert and exceptional performance: evidence of maximal adaptation to task constraints. Annu. Rev. Psychol. 47, 273–305. 10.1146/annurev.psych.47.1.273 15012483

[ref55] Eysenck M. W. Derakshan N. Santos R. Calvo M. G. (2007). Anxiety and cognitive performance: attentional control theory. Emotion 7, 336–353. 10.1037/1528-3542.7.2.336, PMID: 17516812

[ref57] Filimon F. Nelson J. D. Hagler D. J. Sereno M. I. (2007). Human cortical representations for reaching: mirror neurons for execution, observation, and imagery. NeuroImage 37, 1315–1328. 10.1016/j.neuroimage.2007.06.008, PMID: 17689268 PMC2045689

[ref58] Fitts P. M. Posner M. I. (1967). Human performance. Oxford, England: Brooks/Cole.

[ref59] Fleming K. K. Bandy C. L. Kimble M. O. (2010). Decisions to shoot in a weapon identification task: the influence of cultural stereotypes and perceived threat on false positive errors. Soc. Neurosci. 5, 201–220. 10.1080/17470910903268931, PMID: 19813139 PMC4214075

[ref60] Francois C. Schön D. (2011). Musical expertise boosts implicit learning of both musical and linguistic structures. Cereb. Cortex 21, 2357–2365. 10.1093/cercor/bhr022, PMID: 21383236

[ref61] Gallese V. Fadiga L. Fogassi L. Rizzolatti G. (1996). Action recognition in the premotor cortex. Brain 119, 593–609. 10.1093/brain/119.2.593, PMID: 8800951

[ref62] Gallivan J. P. Culham J. C. (2015). Neural coding within human brain areas involved in actions. Curr. Opin. Neurobiol. 33, 141–149. 10.1016/j.conb.2015.03.012, PMID: 25876179

[ref63] Galton F. (1869). Hereditary genius: An inquiry into its laws and consequences. London, Great Britain: Macmillan 1–390. 10.1037/13474-000

[ref64] Gladwell M. (2008). Outliers: The story of success. New York: Hachette Book Group.

[ref65] Gobet F. Simon H. A. (1998). Expert chess memory: revisiting the chunking hypothesis. Memory 6, 225–255. 10.1080/741942359, PMID: 9709441

[ref66] Grèzes J. Decety J. (2001). Functional anatomy of execution, mental simulation, observation, and verb generation of actions: a meta-analysis. Hum. Brain Mapp. 21, 1–19. 10.1002/1097-0193(200101)12:1<1::AID-HBM10>3.0.CO;2-V PMC6872039 11198101

[ref67] Hajcak G. McDonald N. Simons R. F. (2003). Anxiety and error-related brain activity. Biol. Psychol. 64, 77–90. 10.1016/S0301-0511(03)00103-0, PMID: 14602356

[ref68] Haller J. Raczkevy-Deak G. Gyimesine K. P. Szakmary A. Farkas I. Vegh J. (2014). Cardiac autonomic functions and the emergence of violence in a highly realistic model of social conflict in humans. Front. Behav. Neurosci. 8:364. 10.3389/fnbeh.2014.00364, PMID: 25374519 PMC4204534

[ref69] Hammerness K. Darling-Hammond L. Bransford J. Berliner D. Cochran-Smith M. McDonald M. (2007). “How teachers learn and develop” in Preparing teachers for a changing world. eds. Darling-Hammond L. Bransford J. LePage P. Hammerness K. Duffy H. (Hoboken, NJ: John Wiley & Sons), 390–441.

[ref70] Hammerness K. Darling-Hammond L. Grossman P. Rust F. Shulman L. (2005). “The design of teacher education programs” in Preparing teachers for a changing world. eds. Darling-Hammond L. Bransford J. LePage P. Hammerness K. Duffy H. (Hoboken, NJ: John Wiley & Sons), 390–441.

[ref71] Hänggi J. Koeneke S. Bezzola L. Jäncke L. (2010). Structural neuroplasticity in the sensorimotor network of professional female ballet dancers. Hum. Brain Mapp. 31, 1196–1206. 10.1002/hbm.20928, PMID: 20024944 PMC6870845

[ref72] Harmon-Jones E. Gable P. A. Peterson C. K. (2010). The role of asymmetric frontal cortical activity in emotion-related phenomena: a review and update. Biol. Psychol. 84, 451–462. 10.1016/j.biopsycho.2009.08.010, PMID: 19733618

[ref73] Hebb D. O. (1949). The organisation of behavior: A neuropsychological theory. New York: John Wiley & Sons, Inc.

[ref74] Heiberger L. Maurer C. Amtage F. Mendez-Balbuena I. Schulte-Mönting J. Hepp-Reymond M. C.. (2011). Impact of a weekly dance class on the functional mobility and on the quality of life of individuals with Parkinson’s disease. Front. Aging Neurosci. 3:14. 10.3389/fnagi.2011.00014, PMID: 22013420 PMC3189543

[ref75] Hemeren P. E. Thill S. (2011). Deriving motor primitives through action segmentation. Front. Psychol. 1, 1–11. 10.3389/fpsyg.2010.00243 PMC3153847 21833296

[ref76] Hird J. S. Landers D. M. Thomas J. R. Horan J. J. (1991). Physical practice is superior to mental practice in enhancing cognitive and motor task performance. J. Sport Exerc. Psychol. 13, 281–293. 10.1123/jsep.13.3.281

[ref77] Hope L. (2016). Evaluating the effects of stress and fatigue on police officer response and recall: a challenge for research, training, practice and policy. J. Appl. Res. Mem. Cogn. 5, 239–245. 10.1016/j.jarmac.2016.07.008

[ref78] Hope L. Blocksidge D. Gabbert F. Sauer J. D. Lewinski W. Mirashi A.. (2016). Memory and the operational witness: police officer recall of firearms encounters as a function of active response role. Law Hum. Behav. 40, 23–35. 10.1037/lhb0000159, PMID: 26436335

[ref500] Iacoboni M. Koski L. M. Brass M. Bekkering H. Woods R. P. Dubeau M.-C.. (2001). Reafferent copies of imitated actions in the right superior temporal cortex. Proc. Natl. Acad. Sci. 98, 13995–13999. PMID: 11717457 10.1073/pnas.241474598 PMC61155

[ref79] Jameson J. P. Mendes W. B. Blackstock E. Schmader T. (2010). Turning the knots in your stomach into bows: reappraising arousal improves performance on the GRE. J. Exp. Soc. Psychol. 46, 208–212. 10.1016/j.jesp.2009.08.015, PMID: 20161454 PMC2790291

[ref80] Joddrell P. Astell A. J. (2016). Studies involving people with dementia and touchscreen technology: a literature review. JMIR Rehabil. Assist. Technol. 3:e10. 10.2196/rehab.5788, PMID: 28582254 PMC5454556

[ref81] Johnson R. R. Stone B. T. Miranda C. M. Vila B. James L. James S. M.. (2014). Identifying psychophysiological indices of expert vs. novice performance in deadly force judgment and decision making. Front. Hum. Neurosci. 8:512. 10.3389/fnhum.2014.00512, PMID: 25100966 PMC4107851

[ref501] Kahneman D. (2003). A perspective on judgement and choice: mapping bounded rationality. Am. Psychol. 58, 697–720. 10.1037/0003-066X.58.9.697 14584987

[ref82] Karni A. Meyer G. Jezzard P. Adams M. M. Turner R. Ungerleider L. G. (1995). Functional MRI evidence for adult motor cortex plasticity during motor skill learning. Nature 377, 155–158. 10.1038/377155a0, PMID: 7675082

[ref83] Kilner J. M. Lemon R. N. (2013). What we know currently about mirror neurons. Curr. Biol. 23, R1057–R1062. 10.1016/j.cub.2013.10.051, PMID: 24309286 PMC3898692

[ref84] KingJ. B.JonesK. G.GoldbergE.RollinsM.MacNameeK.MoffitC.. (2019). Increased functional connectivity after listening to favored music in adults with Alzheimer dementia. J. Prev. Alzheimers Dis. 6, 56–62. 10.14283/jpad.2018.19, PMID: 30569087

[ref85] Klein G. A. (1989). Strategies of decision making. Yellow Springs, OH: Klein Associates Inc.

[ref86] Klein G. A. (1993). A recognition-primed decision (RPD) model of rapid decision making. New York: Ablex Publishing Corporation, 138–147.

[ref87] Klein G. A. (2017). Sources of power: How people make decisions. Boston, MA: MIT press.

[ref88] Klinger D. A. Brunson R. K. (2009). Police officers’ perceptual distortions during lethal force situations: informing the reasonableness standard. Criminol. Public Policy 8, 117–140. 10.1111/j.1745-9133.2009.00537.x

[ref89] Koch G. Ponzo V. Di Lorenzo F. Caltagirone C. Veniero D. (2013). Hebbian and anti-Hebbian spike-timing-dependent plasticity of human cortico-cortical connections. J. Neurosci. 33, 9725–9733. 10.1523/JNEUROSCI.4988-12.2013, PMID: 23739969 PMC6619701

[ref90] Krameddine Y. I. DeMarco D. Hassel R. Silverstone P. H. (2013). A novel training program for police officers that improves interactions with mentally ill individuals and is cost-effective. Front. Psych. 4, 1–10. 10.3389/fpsyt.2013.00009 PMC3600939 23515226

[ref91] Lansing K. Amen D. G. Hanks C. Rudy L. (2005). High–resolution brain SPECT imaging and eye movement desensitization and reprocessing in police officers with PTSD. J. Neuropsychiatry Clin. Neurosci. 17, 526–532. 10.1176/jnp.17.4.526, PMID: 16387993

[ref92] Lehrer P. M. Gevirtz R. (2014). Heart rate variability biofeedback: how and why does it work? Front. Psychol. 5:756. 10.3389/fpsyg.2014.00756, PMID: 25101026 PMC4104929

[ref93] Lerner Y. Honey C. J. Silbert L. J. Hasson U. (2011). Topographic mapping of a hierarchy of temporal receptive windows using a narrated story. J. Neurosci. 31, 2906–2915. 10.1523/JNEUROSCI.3684-10.2011, PMID: 21414912 PMC3089381

[ref94] Lewinski W. J. Dysterheft J. L. Priem M. M. Pettitt R. W. (2016). Police officers’ actual vs. recalled path of travel in response to a threatening traffic stop scenario. Police Pract. Res. 17, 51–67. 10.1080/15614263.2014.959950

[ref95] Lovallo W. R. (2016). Stress and health: Biological and psychological interactions. 3rd Edn. Thousand Oaks, CA: SAGE.

[ref96] Makin D. A. (2016). A descriptive analysis of a problem-based learning police academy. Interdiscip. J. Probl. Based Learn. 10, 1–15. 10.7771/1541-5015.1544

[ref97] Mattar A. A. Gribble P. L. (2005). Motor learning by observing. Neuron 46, 153–160. 10.1016/j.neuron.2005.02.009, PMID: 15820701

[ref98] McCord J. (2003). Cures that harm: unanticipated outcomes of crime prevention programs. Ann. Am. Acad. Pol. Soc. Sci. 587, 16–30. 10.1177/0002716202250781

[ref99] McIntosh G. C. Brown S. H. Rice R. R. Thaut M. H. (1997). Rhythmic auditory-motor facilitation of gait patterns in patients with Parkinson’s disease. J. Neurol. Neurosurg. Psychiatry 62, 22–26. 10.1136/jnnp.62.1.22, PMID: 9010395 PMC486690

[ref100] Moazami-Goudarzi M. Sarnthein J. Michels L. Moukhtieva R. Jeanmonod D. (2008). Enhanced frontal low and high frequency power and synchronization in the resting EEG of parkinsonian patients. NeuroImage 41, 985–997. 10.1016/j.neuroimage.2008.03.032, PMID: 18457962

[ref101] Morgan C. A. III Doran A. Steffian G. Hazlett G. Southwick S. M. (2006). Stress-induced deficits in working memory and visuo-constructive abilities in special operations soldiers. Biol. Psychiatry 60, 722–729. 10.1016/j.biopsych.2006.04.021, PMID: 16934776

[ref102] Morgan C. A. III Hazlett G. Doran A. Garrett S. Hoyt G. Thomas P.. (2004). Accuracy of eyewitness memory for persons encountered during exposure to highly intense stress. Int. J. Law Psychiatry 27, 265–279. 10.1016/j.ijlp.2004.03.004, PMID: 15177994

[ref103] Murphy J. J. (2014). Beyond a split-second: An exploratory study of police use of force and use of force training in Canada (Master’s Thesis). Simon Fraser University Retrieved from Semantic Scholar database.

[ref104] Ness D. Calabrese P. (2016). Stress effects on multiple memory system interactions. Neural Plast. 2016, 1–20. 10.1155/2016/4932128, PMID: 27034845 PMC4807050

[ref105] Neva J. L. Henriques D. Y. P. (2013). Visuomotor adaptation and generalization with repeated and varied training. Exp. Brain Res. 226, 363–372. 10.1007/s00221-013-3444-1, PMID: 23455723

[ref107] Nieuwenhuys A. Caljouw S. R. Leijsen M. R. Schmeits B. A. J. Oudejans R. R. D. (2009). Quantifying police officers’ arrest and self-defense skills: does performance decrease under pressure? Ergonomics 52, 1460–1468. 10.1080/00140130903287981, PMID: 19941180

[ref108] Nieuwenhuys A. Cañal-Bruland R. Oudejans R. R. (2012a). Effects of threat on police officers’ shooting behavior: anxiety, action specificity, and affective influences on perception. Appl. Cogn. Psychol. 26, 608–615. 10.1002/acp.2838

[ref109] Nieuwenhuys A. Oudejans R. R. (2010). Effects of anxiety on handgun shooting behavior of police officers: a pilot study. Anxiety Stress Coping 23, 225–233. 10.1080/10615800902977494, PMID: 19462309

[ref110] Nieuwenhuys A. Oudejans R. R. (2011). Training with anxiety: short-and long-term effects on police officers’ shooting behavior under pressure. Cogn. Process. 12, 277–288. 10.1007/s10339-011-0396-x, PMID: 21431863 PMC3142543

[ref111] Nieuwenhuys A. Savelsbergh G. J. Oudejans R. R. (2012b). Shoot or don’t shoot? Why police officers are more inclined to shoot when they are anxious. Emotion 12, 827–833. 10.1037/a0025699 22023363

[ref112] Nieuwenhuys A. Savelsbergh G. J. Oudejans R. R. (2015). Persistence of threat-induced errors in police officers’ shooting decisions. Appl. Ergon. 48, 263–272. 10.1016/j.apergo.2014.12.006, PMID: 25683553

[ref113] Nieuwenhuys A. Weber J. van der Hoeve R. Oudejans R. R. (2017). Sitting duck or scaredy-cat? Effects of shot execution strategy on anxiety and police officers’ shooting performance under high threat. Leg. Criminol. Psychol. 22, 274–287. 10.1111/lcrp.12099

[ref114] Oosterhof N. N. Tipper S. P. Downing P. E. (2013). Crossmodal and action-specific: neuroimaging the human mirror neuron system. Trends Cogn. Sci. 17, 311–318. 10.1016/j.tics.2013.04.012, PMID: 23746574

[ref115] Oudejans R. R. D. (2008). Reality based practice under pressure improves handgun shooting performance of police officers. Ergonomics 51, 261–273. 10.1080/00140130701577435, PMID: 17896226

[ref116] Oudejans R. R. Pijpers J. R. (2009). Training with anxiety has a positive effect on expert perceptual–motor performance under pressure. Q. J. Exp. Psychol. 62, 1631–1647. 10.1080/17470210802557702 19123115

[ref117] Oudejans R. R. Pijpers J. R. (2010). Training with mild anxiety may prevent choking under higher levels of anxiety. Psychol. Sport Exerc. 11, 44–50. 10.1016/j.psychsport.2009.05.002

[ref118] Page J. W. Asken M. J. Zwemer C. F. Guido M. (2016). Brief mental skills training improves memory and performance in high stress police cadet training. J. Police Crim. Psychol. 31, 122–126. 10.1007/s11896-015-9171-8

[ref119] Pijpers J. R. Oudejans R. R. Bakker F. C. Beek P. J. (2006). The role of anxiety in perceiving and realizing affordances. Ecol. Psychol. 18, 131–161. 10.1207/s15326969eco1803_1

[ref120] Planche K. Chan J. Di Nota P. M. Beston B. Boychuk E. C. Collins P.. (2019). Diurnal cortisol variation according to high risk occupational speciality within police: comparisons between frontline, tactical officers, and the general population. J. Occup. Environ. Med. 61, e260–e265. 10.1097/JOM.0000000000001591, PMID: 31167225

[ref121] Porro C. A. Facchin P. Fusi S. Dri G. Fadiga L. (2007). Enhancement of force after action observation: behavioural and neurophysiological studies. Neuropsychologia 45, 3114–3121. 10.1016/j.neuropsychologia.2007.06.016, PMID: 17681358

[ref122] Pryor K. (1999). Don’t shoot the dog–the new art of teaching and training (Revised). New York, NY: Bantam Books.

[ref123] Pynn L. K. DeSouza J. F. X. (2013). The function of efference copy signals: implications for symptoms of schizophrenia. Vis. Res. 76, 124–133. 10.1016/j.visres.2012.10.019 23159418

[ref124] Rauste-von Wright M.-L. Wright J. (1994). Oppiminen ja koulutus [Learning and education]. Juva: WSOY:n graafisetlaitokset.

[ref125] Reijnders J. S. Ehrt U. Weber W. E. Aarsland D. Leentjens A. F. (2008). A systematic review of prevalence studies of depression in Parkinson’s disease. Mov. Disord. 23, 183–189. 10.1002/mds.21803 17987654

[ref126] Rizzolatti G. Craighero L. (2004). The mirror-neuron system. Annu. Rev. Neurosci. 27, 169–192. 10.1146/annurev.neuro.27.070203.144230, PMID: 15217330

[ref127] Rizzolatti G. Fadiga L. Gallese V. Fogassi L. (1996). Premotor cortex and the recognition of motor actions. Cogn. Brain Res. 3, 131–141. 10.1016/0926-6410(95)00038-0, PMID: 8713554

[ref128] Rizzolatti G. Luppino G. Matelli M. (1998). The organization of the cortical motor system: new concepts. Electroencephalogr. Clin. Neurophysiol. 106, 283–296. 10.1016/S0013-4694(98)00022-4, PMID: 9741757

[ref129] Ropo E. (1991). Opettajaeksperttiyden kehittyminen–tutkimustuloksia ja näkökulmia [Developing teacher expertise–research results and perspectives]. Aikuiskasvatus 11, 153–163.

[ref130] Roth M. Decety J. Raybaudi M. Massarelli R. Delon-Martin C. Segebarth C.. (1996). Possible involvement of primary motor cortex in mentally simulated movement: a functional magnetic resonance imaging study. Neuroreport 7, 1280–1284. 10.1097/00001756-199605170-00012, PMID: 8817549

[ref131] Sakai K. Kitaguchi K. Hikosaka O. (2003). Chunking during human visuomotor sequence learning. Exp. Brain Res. 152, 229–242. 10.1007/s00221-003-1548-8, PMID: 12879170

[ref132] Salas E. Bowers C. A. Cannon-Bowers J. A. (1995). Military team research: 10 years of progress. Mil. Psychol. 7, 55–75. 10.1207/s15327876mp0702_2

[ref133] Salomonczyk D. Cressman E. K. Henriques D. Y. P. (2011). Proprioceptive recalibration following prolonged training and increasing distortions in visuomotor adaptation. Neuropsychologia 49, 3053–3062. 10.1016/j.neuropsychologia.2011.07.006, PMID: 21787794

[ref134] Schmidt R. A. Lee T. D. Winstein C. Wulf G. Zelaznik H. N. (2019). Motor control and learning: A behavioral emphasis (6th Ed.). Champaign, IL: Human kinetics.

[ref135] Schwabe L. Wolf O. T. (2013). Stress and multiple memory systems: from ‘thinking’ to ‘doing’. Trends Cogn. Sci. 17, 60–68. 10.1016/j.tics.2012.12.001, PMID: 23290054

[ref136] Sherwood D. E. Lee T. D. (2003). Schema theory: critical review and implications for the role of cognition in a new theory of motor learning. Res. Q. Exerc. Sport 74, 376–382. 10.1080/02701367.2003.10609107, PMID: 14768838

[ref137] Shmuelof L. Krakauer J. W. Mazzoni P. (2012). How is a motor skill learned? Change and invariance at the levels of task success and trajectory control. J. Neurophysiol. 108, 578–594. 10.1152/jn.00856.2011, PMID: 22514286 PMC3404800

[ref138] Simon H. A. Chase W. G. (1973). Skill in chess. Am. Sci. 61, 394–403.

[ref139] Soikkeli R. Partanen J. Soininen H. Pääkkönen A. Riekkinen P. Sr. (1991). Slowing of EEG in Parkinson’s disease. Electroencephalogr. Clin. Neurophysiol. 79, 159–165. 10.1016/0013-4694(91)90134-P, PMID: 1714807

[ref140] Song S. Miller K. D. Abbott L. F. (2000). Competitive Hebbian learning through spike-timing-dependent synaptic plasticity. Nat. Neurosci. 3, 919–926. 10.1038/78829, PMID: 10966623

[ref141] Stanny C. J. Johnson T. C. (2000). Effects of stress induced by a simulated shooting on recall by police and citizen witnesses. Am. J. Psychol. 113, 359–386. 10.2307/1423364, PMID: 10997233

[ref502] Stanovich K. E. West R. F. (2000). Individual differences in reasoning:Implications for the rationality debate. Behav. Brain Sci. 23, 645–665. PMID: 11301544 10.1017/s0140525x00003435

[ref142] Stevenson R. A. Geoghegan M. L. James T. W. (2007). Superadditive BOLD activation in superior temporal sulcus with threshold non-speech objects. Exp. Brain Res. 179, 85–95. 10.1007/s00221-006-0770-6, PMID: 17109108

[ref143] Taverniers J. Taylor M. K. Sweets T. (2013). Delayed memory effects after intense stress in special forces candidates: exploring path processes between cortisol secretion and memory recall. Stress 16, 311–320. 10.3109/10253890.2012.721824 22900536

[ref144] Thaut M. H. (2015). “The discovery of human auditory–motor entrainment and its role in the development of neurologic music therapy” in Progress in brain research. eds. Altenmüller E. Finger S. Boller F. (Amsterdam, NL: Elsevier), 253–266. 10.1016/bs.pbr.2014.11.030 25725919

[ref145] Thaut M. H. McIntosh G. C. Rice R. R. Miller R. A. Rathbun J. Brault J. M. (1996). Rhythmic auditory stimulation in gait training for Parkinson’s disease patients. Mov. Disord. 11, 193–200. 10.1002/mds.870110213, PMID: 8684391

[ref146] Thayer J. F. Sternberg E. (2006). Beyond heart rate variability: vagal regulation of allostatic systems. Ann. N. Y. Acad. Sci. 1088, 361–372. 10.1196/annals.1366.014, PMID: 17192580

[ref147] Tiippana K. (2006). “Moniaistinen havaitseminen [Multisensory perception]” in Mieli ja Aivot: Kongnitiivisen neurotieteen oppikirja. [Mind and brain: Cognitive science handbook]. eds. Hämäläinen H. Laine M. Aaltonen O. Revonsuo A. (Center for Cognitive Neuroscience, University of Turku). Turku, FI: Gummerus Kirjapaino), 177–184.

[ref148] Tippett W. J. Sergio L. E. (2006). Visuomotor integration is impaired in early stage Alzheimer’s disease. Brain Res. 1102, 92–102. 10.1016/j.brainres.2006.04.049, PMID: 16797495

[ref149] Toiskallio J. Mäkinen J. (2009). Military pedagogy: Military and action competence theory and practice. Helsinki: Department of Management and Military Education. MPKK Publication Series. Edita Prima.

[ref150] Tremblay P.-L. Bedard M.-A. Langlois D. Blanchet P. J. Lemay M. Parent M. (2010). Movement chunking during sequence learning is a dopamine-dependant process: a study conducted in Parkinson’s disease. Exp. Brain Res. 205, 375–385. 10.1007/s00221-010-2372-6, PMID: 20680249

[ref151] Varila J. Rekola H. (2003). Mitä on työssä oppiminen: teoreettisia ja empiirisiä näkökulmia työssä oppimiseen [What is learning at work: Theoretical and empirical perspectives on learning at work]. Joensuu: Joensuun yliopisto.

[ref152] Ventura R. E. Balcer L. J. Galetta S. L. Rucker J. C. (2016). Ocular motor assessment in concussion: current status and future directions. J. Neurol. Sci. 361, 79–86. 10.1016/j.jns.2015.12.010, PMID: 26810521

[ref153] Vicente K. J. Wang J. H. (1998). An ecological theory of expertise effects in memory recall. Psychol. Rev. 105, 33–57. 10.1037/0033-295X.105.1.33, PMID: 9450371

[ref154] Vickers J. N. Lewinski W. (2012). Performing under pressure: gaze control, decision making and shooting performance of elite and rookie police officers. Hum. Mov. Sci. 31, 101–117. 10.1016/j.humov.2011.04.004, PMID: 21807433

[ref155] Weinberg A. Meyer A. Hale-Rude E. Perlman G. Kotov R. Klein D. N.. (2016). Error-related negativity (ERN) and sustained threat: conceptual framework and empirical evaluation in an adolescent sample. Psychophysiology 53, 372–385. 10.1111/psyp.12538, PMID: 26877129 PMC4756390

[ref156] Werner S. Noppeney U. (2010). Superadditive responses in superior temporal sulcus predict audiovisual benefits in object categorization. Cereb. Cortex 20, 1829–1842. 10.1093/cercor/bhp248, PMID: 19923200

[ref157] Westheimer O. McRae C. Henchcliffe C. Fesharaki A. Glazman S. Ene H.. (2015). Dance for PD: a preliminary investigation of effects on motor function and quality of life among persons with Parkinson’s disease (PD). J. Neural Transm. 122, 1263–1270. 10.1007/s00702-015-1380-x, PMID: 25836752

[ref158] Yager L. M. Garcia A. F. Wunsch A. M. Ferguson S. M. (2015). The ins and outs of the striatum: role in drug addiction. Neuroscience 301, 529–541. 10.1016/j.neuroscience.2015.06.033, PMID: 26116518 PMC4523218

[ref159] Yerkes R. M. Dodson J. D. (1908). The relation of strength of stimulus to rapidity of habit-formation. J. Comp. Neurol. Psychol. 18, 459–482. 10.1002/cne.920180503

[ref160] Yin H. H. Knowlton B. J. (2006). The role of the basal ganglia in habit formation. Nat. Rev. Neurosci. 7, 464–476. 10.1038/nrn1919, PMID: 16715055

[ref161] Yue G. Cole K. J. (1992). Strength increases from the motor program: comparison of training with maximal voluntary and imagined muscle contractions. J. Neurophysiol. 67, 1114–1123. 10.1152/jn.1992.67.5.1114, PMID: 1597701

[ref162] Yuille J. C. Davies G. Gibling F. Marxsen D. Porter S. (1994). Eyewitness memory of police trainees for realistic role plays. J. Appl. Psychol. 79, 931–936. 10.1037/0021-9010.79.6.931

[ref163] Zacks J. M. Kumar S. Abrams R. A. Mehta R. (2009b). Using movement and intentions to understand human activity. Cognition 112, 201–216. 10.1016/j.cognition.2009.03.007 19497569

[ref164] Zacks J. M. Sargent J. Q. (2010). “Chapter 7–event perception: a theory and its application to clinical neuroscience” in Introduction to functional magnetic resonance imaging. 1st Edn. Vol. 53. ed. Ross B. (Burlington: Elsevier Inc.), 253–299.

[ref165] Zacks J. M. Speer N. K. Reynolds J. R. (2009a). Segmentation in reading and film comprehension. J. Exp. Psychol. Gen. 138, 307–327. 10.1037/a0015305 19397386 PMC8710938

[ref166] Zacks J. M. Speer N. K. Swallow K. M. Maley C. J. (2010). The brain’s cutting-room floor: segmentation of narrative cinema. Front. Hum. Neurosci. 4, 1–15. 10.3389/fnhum.2010.00168 20953234 PMC2955413

[ref167] Zacks J. M. Swallow K. M. Vettel J. M. McAvoy M. P. (2006). Visual motion and the neural correlates of event perception. Brain Res. 1076, 150–162. 10.1016/j.brainres.2005.12.122, PMID: 16473338

[ref168] Zalla T. Pradat-Diehl P. Sirigu A. (2003). Perception of action boundaries in patients with frontal lobe damage. Neuropsychologia 41, 1619–1627. 10.1016/S0028-3932(03)00098-8, PMID: 12887987

[ref169] Zalla T. Verlut I. Franck N. Puzenat D. Sirigu A. (2004). Perception of dynamic action in patients with schizophrenia. Psychiatry Res. 128, 39–51. 10.1016/j.psychres.2003.12.026, PMID: 15450913

